# Sex-specific disruptions in PKC**γ** signaling in a mouse model of spinocerebellar ataxia type 14

**DOI:** 10.1172/jci.insight.192155

**Published:** 2026-04-02

**Authors:** Sarah A. Wolfe, Yuliang Ma, Tomer M. Yaron-Barir, Carly Chang, Caila A. Pilo, Majid Ghassemian, Amanda J. Roberts, Sang Ryeul Lee, Benjamin A. Henson, Kristen Jepsen, Jared L. Johnson, Lewis C. Cantley, Susan S. Taylor, George Gorrie, Alexandra C. Newton

**Affiliations:** 1 Department of Pharmacology, UCSD, La Jolla, California, USA.; 2 Dana-Farber Cancer Institute, Harvard Medical School, Boston, Massachusetts, USA.; 3 Department of Pediatrics, Boston Children’s Hospital, Harvard Medical School, Boston, Massachusetts, USA.; 4 Department of Chemistry and Biochemistry, Biomolecular and Proteomics Mass Spectrometry Facility, UCSD, La Jolla, California, USA.; 5 Animal Models Core Facility, The Scripps Research Institute, La Jolla, California, USA.; 6 The UCSD Transgenic Mouse Core, and; 7 Institute for Genomic Medicine, UCSD, La Jolla, California, USA.; 8 Department of Cell Biology, Harvard Medical School, Boston, Massachusetts, USA.; 9 Queen Elizabeth University Hospital, Glasgow, United Kingdom.

**Keywords:** Cell biology, Neuroscience, Neurodegeneration, Neurological disorders, Protein kinases

## Abstract

Spinocerebellar ataxia type 14 (SCA14) is an autosomal dominant neurodegenerative disease caused by mutations in the gene encoding protein kinase C γ (PKCγ), a Ca^2+^- and diacylglycerol-dependent Ser/Thr kinase dominantly expressed in cerebellar Purkinje cells. These mutations impair autoinhibitory constraints to increase the basal activity of the kinase, resulting in deficits in the cerebellum that are not observed upon simple deletion of the gene, and severe ataxia. To better understand the impact of aberrant PKCγ signaling in disease pathology, we developed a knockin murine model of the SCA14 mutation ΔF48 in PKCγ. This fully penetrant mutation is severe in humans and is mechanistically informative, as it has high basal activity but is unresponsive to agonist stimulation. Genetic, behavioral, and molecular testing revealed that ΔF48 PKCγ mice have ataxia-related phenotypes and an altered cerebellar phosphoproteome driven primarily by enhanced Ca^2+^/calmodulin-dependent kinase 2 signaling, effects that were more severe in male mice. Analysis of existing human data revealed that SCA14 has a significantly earlier age of onset for males compared with females. Data from this clinically relevant mutation suggested that enhanced basal activity of PKCγ is sufficient to cause ataxia and that treatment strategies to modulate aberrant PKCγ may be particularly beneficial in males.

## Abstract 2

New mouse model of Spinocerebellar Ataxia Type 14 containing a clinically relevant mutation in PKCγ identified underlying drivers of the disease and neuroprotection in females.

## Introduction

The neurodegenerative disease spinocerebellar ataxia type 14 (SCA14) is caused by missense mutations in the Ca^2+^- and diacylglycerol-dependent (DG-dependent) Ser/Thr kinase, protein kinase C γ (PKCγ) (1). SCAs are a group of autosomal dominant diseases characterized by cerebellar dysfunction, progressive ataxia, loss of motor coordination, increased disease severity with age, and cognitive-affective disturbances (2–15). SCAs are genetically heterogeneous, with close to 50 genetic causes for the disease identified (9, 16). Germline missense mutations in PKCγ were first identified 2 decades ago and there are now over 75 known mutations in PKCγ that define SCA14 (1, 2, 17). The majority of SCAs are driven by dysregulated Ca^2+^ homeostasis, including mutations in genes encoding the primary inositol 1,4,5-trisphosphate (IP_3_) receptor in the brain (SCA15, -16, and -29), Ca^2+^ channel subunits (SCA25 and -26), and voltage-gated K^+^ channels that impact voltage-gated Ca^2+^ channel function (SCA13, -19, and -22). As a key transducer of Ca^2+^ signals, aberrant PKCγ signaling is associated with most SCAs (18–21). Understanding the pathology of PKCγ mutations in SCA14 may open avenues for therapeutically targeting SCAs in general, particularly given the druggability of protein kinases.

PKC isozymes play key roles in normal brain physiology by regulating neuronal functions such as synapse morphology, receptor turnover, and cytoskeletal integrity. As with all conventional PKC isozymes, PKCγ is tightly regulated and is maintained in an “off” autoinhibited state by a set of N-terminal regulatory domains that prevent the C-terminal kinase domain from phosphorylating its substrates (20–23). Notably, the substrate-binding site of PKCγ binds an autoinhibitory pseudosubstrate segment that maintains PKCγ in an inactive conformation; binding of the second messengers Ca^2+^ and DG releases the pseudosubstrate, allowing PKC to adopt the “on” and signaling-competent conformation. These second messengers are produced by phospholipase C–catalyzed (PLC-catalyzed) hydrolysis of phosphatidylinositol 4,5-bisphosphate (PIP_2_) to produce IP_3_, which induces intracellular Ca^2+^ release, and the allosteric activator DG. Binding of Ca^2+^ to the C2 domain recruits PKCγ to the plasma membrane, where it binds the membrane-embedded DG via the C1B domain, to relieve autoinhibition and permit efficient substrate phosphorylation (20–26). Metabolism of DG and a reduction in cytosolic Ca^2+^ cause PKCγ to re-autoinhibit and adopt the off state (27, 28). Improperly autoinhibited PKC is degraded by a quality control mechanism that ensures that aberrantly active PKC does not accumulate in the cell (29, 30).

**Figure 1:**
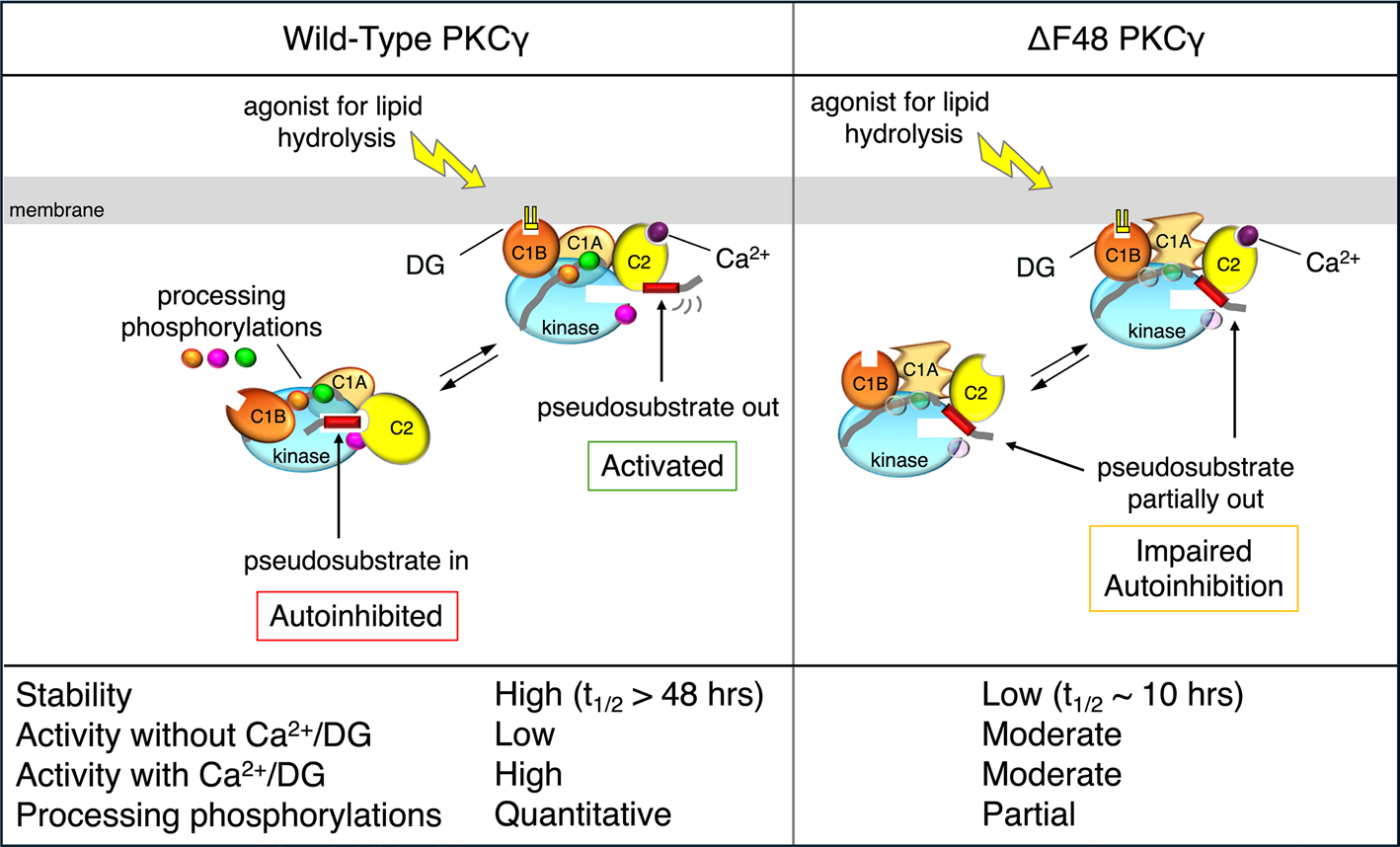
Cartoon summarizing biochemical properties of WT and ΔF48 PKCγ. Left: In the absence of the second messengers diacylglycerol (DG) and Ca^2+^, WT PKC is maintained in an autoinhibited conformation driven by phosphorylation of the hydrophobic motif (green circle) and binding of the pseudosubstrate (red rectangle) to the substrate-binding cavity (20). This species of PKC is phosphatase resistant and stable, with a half-life in cells of over 48 hours. Agonist-evoked generation of second messengers results in reversible Ca^2+^-dependent recruitment to membranes via the C2 domain (yellow oval), followed by binding of DG to the C1B domain (orange oval), events that promote the release of the pseudosubstrate from the substrate-binding cavity to allow substrate binding and downstream signaling. Right: ΔF48 PKCγ is in a “frozen” and partially open conformation with basal activity (approximately 1/3 the *k*_cat_ of Ca^2+^/lipid–stimulated WT enzyme) (31). Although ΔF48 PKCγ can bind second messengers and translocate to membranes, communication of the C1B domain with the pseudosubstrate is lost and thus activity is unresponsive to second messenger binding. This partially open conformation is phosphatase labile and relatively unstable (half-life in cells ~10 hours).

Many SCA14 mutations impair autoinhibitory contacts to increase basal activity of PKCγ, resulting in leaky signaling (20, 31–33). The degree of leaky signaling correlates with disease severity, with mutations that impair autoinhibition the most associated with earliest ages of onset in patients (31). One of the most severe variants, deletion of Phe48 (ΔF48) in PKCγ, is in the C1A domain immediately following the pseudosubstrate domain. ΔF48 PKCγ has reduced autoinhibition resulting in higher basal activity (corresponding to approximately 30% of the activity of fully activated WT PKCγ both vis-à-vis pure protein and activity in cells monitored with biosensors) but also has uncoupled communication between the pseudosubstrate and the kinase domain, making it unresponsive to DG/Ca^2+^ (pure protein) or agonist stimulation (in cells). This slightly open conformation sensitizes ΔF48 PKCγ to quality control degradation, reflected by a 5-fold increase in basal turnover rate compared with WT PKCγ (31). It is noteworthy that although basal turnover is accelerated, phorbol ester–induced downregulation, which requires an intact C1A domain, is abolished; this downregulation occurs by a separate mechanism (34). The unique unresponsiveness of ΔF48 PKCγ to agonist-evoked activation (Figure 1) suggests that the unregulated leaky basal signaling of this SCA14 variant is necessary and sufficient to drive the pathology (20, 31, 32).

PKCγ is dominantly expressed in Purkinje cells (33, 35–39), a major cell type in the cerebellum. Purkinje cells are GABAergic neurons that form highly branched dendritic trees innervated throughout the cerebellar layers (molecular layer [ML], Purkinje cell layer [PCL], and granular layer [GL]) (40–43). PKCγ signaling plays an important role in Purkinje cell function, formation, and innervation in the cerebellum, and disruptions in proper Purkinje cell development occur when PKC activity is dysregulated (33, 35–39, 44–49). Other PKC isoforms are also present in Purkinje cells, including α, δ, and η (36, 50, 51). SCA pathogenesis can be driven by progressive neurodegeneration of Purkinje cells, or by dysfunction and altered firing patterns of Purkinje cells that disrupt cerebellar signaling causing motor dysfunction (6, 7, 33, 43–48, 52 , 53, 54). It is noteworthy that PKCγ-knockout mice lack evident neurodegeneration and only present mild ataxia, revealing that it is not the absence of PKCγ that drives neurodegeneration in SCA14, but the presence of aberrant PKCγ and aberrant signaling (33, 37, 49, 55, 56). How this dysregulated PKCγ signaling drives the pathology of SCA14 remains to be elucidated.

Here we have generated a mouse model of SCA14 containing the severe mutation, ΔF48 PKCγ, to better understand how basally active PKCγ drives the pathology of SCA14. The ΔF48 PKCγ mouse displayed ataxia-associated phenotypes that were more severe in males compared with females. These mice displayed significantly reduced PKCγ protein levels with no reduction in PKC substrate phosphorylation, revealing that the constitutive basal activity of ΔF48 PKCγ compensated for reduced PKC levels. Purkinje cell morphology was altered, but no overt neurodegeneration was identified. Phosphoproteomic analysis revealed significant changes in the cerebellum of the ΔF48 PKCγ mice that primarily impacted neuronal and cytoskeletal pathways, consistent with deficits in Purkinje cells. Taken together with existing human data showing an earlier age of onset of SCA14 symptoms in male compared with female patients, our data from the mouse model suggest that this disease is more severe in males compared with females.

## Results

### ΔF48 PKCγ causes sex-specific ataxia-related deficits in motor functions in a mouse model of SCA14.

ΔF48 PKCγ has enhanced basal activity due to impaired autoinhibition, increased turnover due to quality control degradation, resistance to phorbol ester–induced degradation (which requires an intact C1A domain), and insensitivity to DG/Ca^2+^-induced activation (Figure 1) (20, 31). These severe biochemical defects of ΔF48 PKCγ are associated with early age of onset and high disease severity (11, 20, 31). To understand the pathology of ΔF48 PKCγ–induced SCA14, a ΔF48 PKCγ–knockin mouse was generated with CRISPR/Cas9-mediated genome editing methods (Supplemental Figure 1A; supplemental material available online with this article; https://doi.org/10.1172/jci.insight.192155DS1). This mouse model of SCA14 was validated through whole-genome sequencing and off-target analysis (Supplemental Figure 1, B and C). All mice were genotyped (Supplemental Figure 1, D and E).

**Figure 2:**
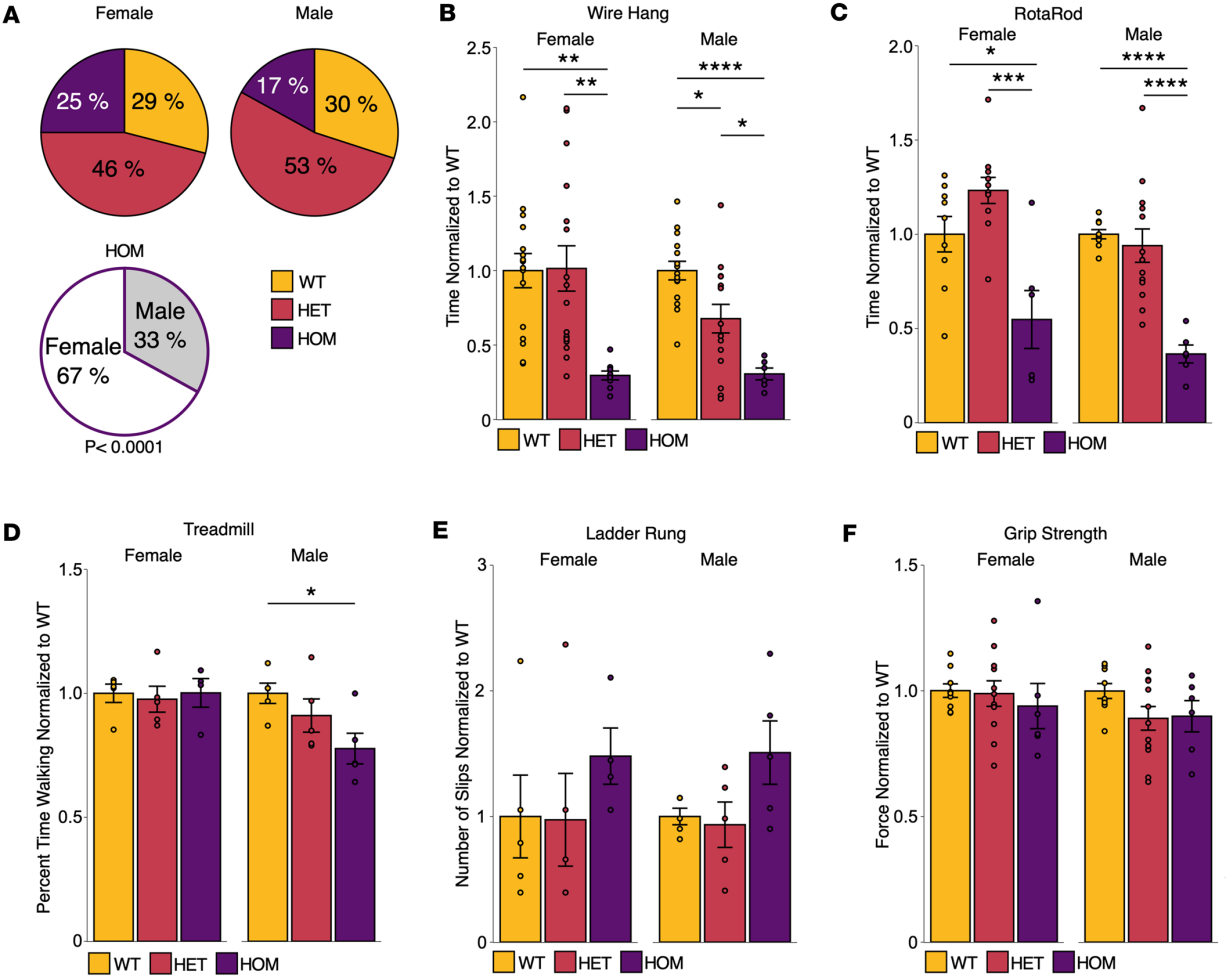
ΔF48 PKCγ causes ataxia-associated deficits in motor function. Viability of SCA14 mice containing the ΔF48 PKCγ mutation was determined by evaluating offspring born from HET crosses. (**A**) Pie charts show genetic inheritance ratios for WT (yellow), HET (red), and HOM (purple) female and male offspring. Significance was determined by the χ^2^ test for expected ratios (*P* < 0.0001, *n* = 182 mice). SCA14 mice were evaluated for ataxic-related behaviors using a battery of motor function test in all genotypes (WT, yellow; HET, red; HOM, purple) and sexes, including (**B**) wire hang test [female (1-way ANOVA: *F*(2,38) = 7.7, *P* = 0.0016; Tukey’s post hoc test: WT vs. HET *P* = 0.99, WT vs. HOM *P* = 0.0032, HET vs. HOM *P* = 0.0027), male (1-way ANOVA: *F*(2,33) = 13, *P* = 0.000069; Tukey’s post hoc test: WT vs. HET *P* = 0.013, WT vs. HOM *P* = 0.000065, HET vs. HOM *P* = 0.033)], (**C**) rotarod test [female (1-way ANOVA, *F*(2,23) = 11, *P* = 0.00040; Tukey’s post hoc test, WT vs. HET *P* = 0.19, WT vs. HOM *P* = 0.017, HET vs. HOM *P* = 0.00026), male (1-way ANOVA, *F*(2,25) = 16, *P* = 0.000033; Tukey’s post hoc test, WT vs. HET *P* = 0.82, WT vs. HOM *P* = 0.000062, HET vs. HOM *P* = 0.000097)], (**D**) treadmill test [female (1-way ANOVA, *F*(2,11) = 0.088, *P* = 0.92), male (1-way ANOVA, *F*(2,12) = 3.8, *P* = 0.052; Tukey’s post hoc test, WT vs. HET *P* = 0.53, WT vs. HOM *P* = 0.044, HET vs. HOM *P* = 0.27)], (**E**) ladder rung test, and (**F**) grip strength test. Data were normalized to WT where multiple cohorts were combined from mice (*n* = 5–16 mice per group; age-matched = 5–10 months). Bar graphs represent mean ± SEM. Significance was determined by 1-way ANOVA with Tukey’s post hoc test. *P < 0.05; **P < 0.01; ***P < 0.001; ***P < 0.0001.

To assess the viability of mice with SCA14 genotypes (heterozygous WT/ΔF48 [HET] or homozygous ΔF48/ΔF48 [HOM]), the birth rates of each genotype were compared from 183 offspring born from HET crosses. Female mice had expected Mendelian inheritance ratios (WT: 29%, HET: 46%, HOM: 25%), whereas male mice had fewer than expected HOM mice born (WT: 30%, HET: 53%, HOM: 17%). In HOM offspring, male (33%) and female (67%) birth ratios were significantly different from expected ratios (Figure 2A). No differences were noted in the health of the adult mice, and no significant differences between genotype were observed in the weight of the adult mice (Supplemental Figure 2). This suggests that male HOM mice have reduced birth rates and possible developmental deficits resulting from aberrant PKCγ signaling (2–8, 15, 37).

A hallmark of SCA14 is cerebellar dysfunction leading to progressive ataxia and loss of motor function (2–15). To evaluate motor function in SCA14 mice with the ΔF48 PKCγ mutation, behavioral tests used to measure motor function, including a wire hang test, a rotarod test, a treadmill walking test, a ladder rung test, and a grip strength test were performed on adult mice over 5 months old. Muscle performance was assessed with the wire hang test by measuring latency to fall time. HOM mice had a markedly reduced latency to fall time by approximately 70% in both sexes compared with WT mice, and a significantly reduced latency to fall time by 70% in females and 54% in males compared with HET mice. Male HET mice also had a significant 32% reduction in hang time compared with WT, whereas female HET mice performed equivalently to WT (Figure 2B). The rotarod test evaluated locomotor coordination and balance by measuring latency to fall from a rotating rod. Both female and male HOM mice had significantly decreased latency to fall times compared with WT (reduced by 45% in females and 64% in males) and HET mice (reduced by 55% in females and 62% in males) (Figure 2C). Deficits in walking were evaluated with a treadmill task in which the mouse’s percentage time spent walking ahead of the bumper/rear of a treadmill was measured. Only male HOM mice were found to have a significantly reduced time (23%) spent walking compared with WT (Figure 2D). The ladder rung test measured skilled walking and coordination by measuring paw slips as mice walk along a horizontal ladder. No significant differences were detected with this test; however, a strong trend toward an increase in the number of slips measured in the male and female HOM mice was observed (Figure 2E). No significant differences were observed in forelimb grip strength between genotypes, although a trend toward a decrease in grip strength was observed in male HET and HOM mice (~10% reduced) (Figure 2F). These behavioral tests indicate that SCA14 mice have ataxia-associated phenotypes that are more severe in males.

### SCA14 mice have altered Purkinje cell morphology.

**Figure 3:**
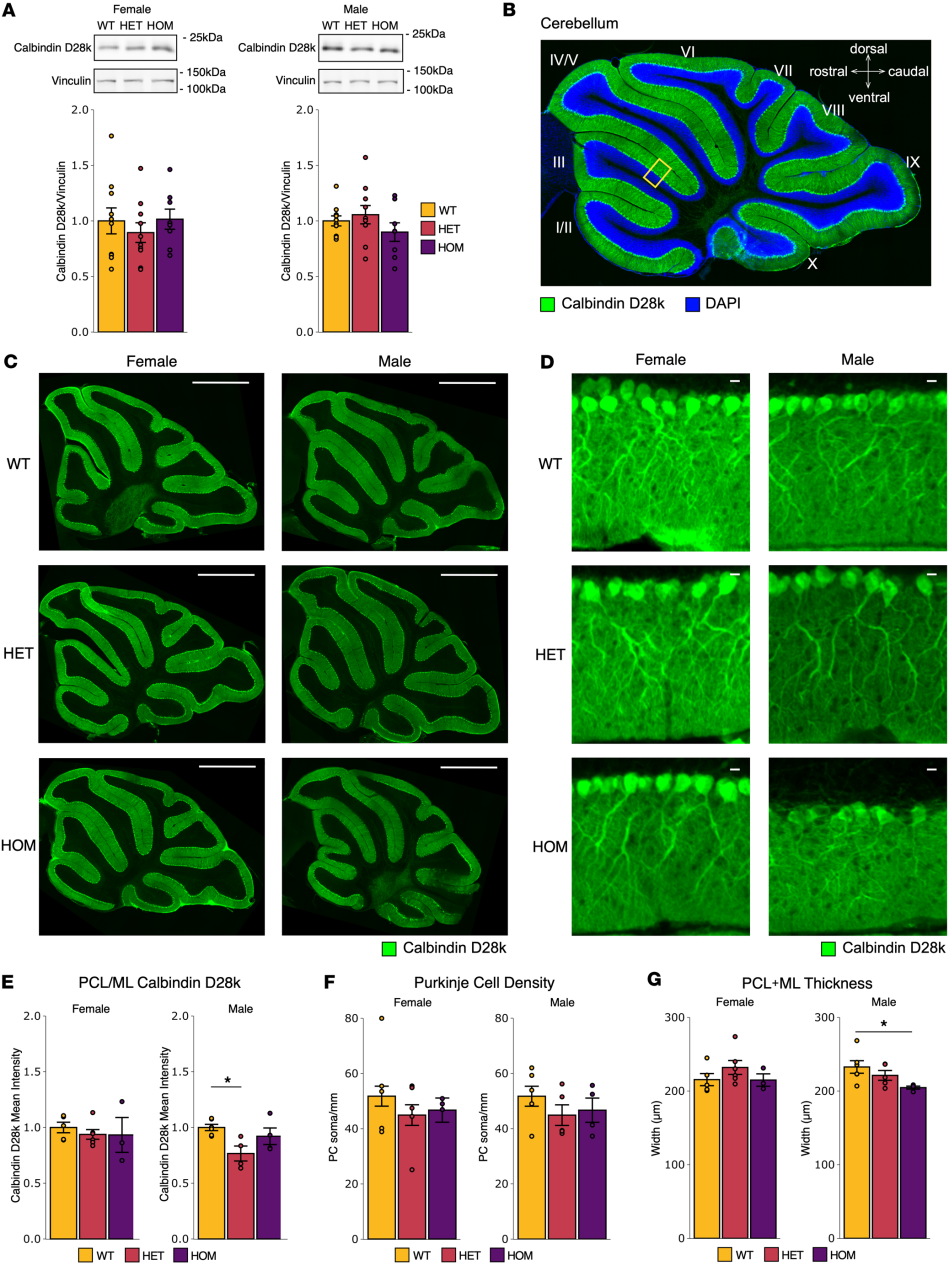
Cerebellum and Purkinje cell morphology are altered in SCA14 mice. The Purkinje cell marker Calbindin D28k was used to assess neuronal morphology in SCA14 mice. (**A**) Western blot analysis of whole cerebellar lysate from SCA14 mice of all genotypes (WT, yellow; HET, red; HOM, purple) and sexes was performed (*n* = 8–10 mice per group). No significant differences were identified. Bar graphs represent mean ± SEM normalized to WT. Fluorescence immunohistochemistry of Calbindin D28k was performed on fixed sagittal brain sections from all genotypes (WT, HET, HOM) and sexes (*n* = 3–6 mice). (**B**) Example image indicates orientation of cerebellum images, and yellow box indicates approximate location of magnified images, shown to the right with labeled Purkinje cell layer (PCL), molecular layer (ML), and granular layer (GL) (Calbindin D28k, green; DAPI, blue). Representative images show intensity and localization of Calbindin D28k (green) in (**C**) whole cerebellum (scale bars: 1 mm) and (**D**) PCL and ML magnification (scale bars: 10 μm). Quantification of PCL and ML of cerebellar images (*n* = 3–6 mice) measured (**E**) the intensity of Calbindin D28k labeling [female (1-way ANOVA, *F*(2,11) = 0.29, *P* = 0.75; Tukey’s post hoc test, WT vs. HET *P* = 0.78, WT vs. HOM *P* = 0.82, HET vs. HOM *P* = 0.99), male (1-way ANOVA, *F*(2,11) = 5.0, *P* = 0.028; Tukey’s post hoc test, WT vs. HET *P* = 0.023, WT vs. HOM *P* = 0.55, HET vs. HOM *P* = 0.18)], (**F**) the linear density of the Purkinje cells, and (**G**) the width of the PCL and ML indicative of dendrite length [female (1-way ANOVA, *F*(2,11) = 1.2, *P* = 0.34; Tukey’s post hoc test, WT vs. HET *P* = 0.40, WT vs. HOM *P* = 1.0, HET vs. HOM *P* = 0.48), male (1-way ANOVA, *F*(2,12) = 3.6, *P* = 0.058; Tukey’s post hoc test, WT vs. HET *P* = 0.49, WT vs. HOM *P* = 0.047, HET vs. HOM *P* = 0.31)]. Bar graphs represent mean ± SEM. Significance was determined by 1-way ANOVA with Tukey’s post hoc test. *P < 0.05.

The cerebellum is a key mediator of motor function, and cerebellar Purkinje cell neurodegeneration and/or dysfunction is a dominant feature of ataxia (33, 40–44, 53, 54). To determine whether adult SCA14 mice with the ΔF48 PKCγ mutation have deficits in cerebellar morphology indicative of ataxic pathology, we assessed the Purkinje cells of the cerebellum using immunofluorescence and Western blot analysis in all genotypes and sexes using the Purkinje cell–specific marker Calbindin D28k. Western blot analysis identified no changes in Calbindin D28k protein abundance in cerebellar homogenate from adult SCA14 mice compared with WT in either male or female mice (Figure 3A), indicative of a lack of overt Purkinje cells neurodegeneration. To further assess the Purkinje cells, immunofluorescent staining of sagittal cerebellar slices (depicted and labeled in Figure 3B) was performed. Overall cerebellar morphology and Calbindin D28k intensity were visually similar in WT and SCA14 mice of both sexes (Figure 3C); however, upon closer inspection of an enlarged image of the PCL and ML (indicated as the yellow box in Figure 3B), variations in Purkinje cells became more apparent. A ΔF48 PKCγ gene dose–dependent reduction (WT > HET > HOM) in dendritic arborization was observed and was particularly evident in male mice, as visualized by a reduction in Purkinje cell dendrite length and Calbindin D28k staining in the ML (Figure 3D). To measure potential differences, we selected the cerebellar layers where Purkinje cells are present (PCL and ML) through all lobules of the cerebellum and analyzed the intensity of Calbindin D28k staining, the density of Purkinje cells, and the thickness of the layers. First, the mean intensity of Calbindin D28k staining was quantified, and no differences were observed in female ΔF48 PKCγ mice compared to WT; however, male ΔF48 PKCγ mice had reduced Calbindin D28k levels compared with WT, with a significant 33% reduction in HET males compared with WT males (Figure 3E). Next, the linear density of Purkinje cell somas was measured per distance along the PCL to identify neurodegeneration of Purkinje cells in SCA14 mice. Although no significant loss of Purkinje cells was found, both male and female SCA14 genotypes trended down in Purkinje cell density (Figure 3F). Lastly, the thickness of the PCL and ML was measured to assess Purkinje cell arborization. The average width of the PCL and ML was measured, and a ΔF48 PKCγ gene dose–dependent reduction in thickness was identified in male mice, with a significant average reduction of approximately 23 μm in HOM compared with WT males, indicating a reduction in dendritic length (Figure 3G). These data suggest that overt neurodegeneration or loss of Purkinje cells was not prevalent in the cerebellum of SCA14 mice; however, Purkinje cells had reduced dendritic length, particularly in male mice.

### SCA14 mice have reduced total PKC levels in the cerebellum.

**Figure 4:**
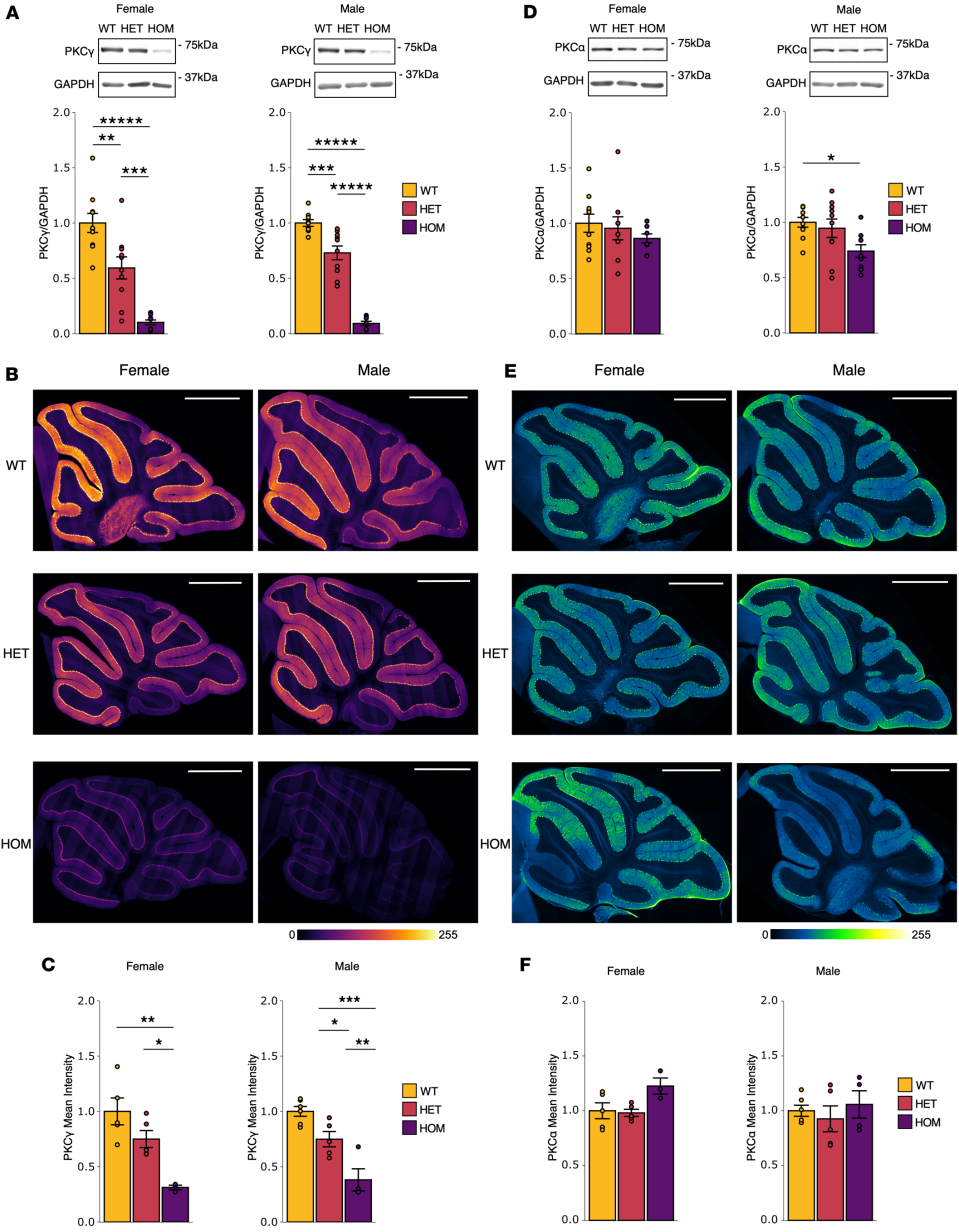
PKCγ and PKCα are gene dose–dependently reduced in SCA14 mice. Western blot analysis of whole cerebellar homogenate from all genotypes (WT, yellow; HET, red; HOM, purple) and sexes (*n* = 8–10 mice per group) was performed to assess cerebellar levels of (**A**) PKCγ [female (1-way ANOVA, *F*(2,26) = 31.14, *P* = 1.26 × 10^–7^; Tukey’s post hoc test, WT vs. HET *P* = 0.0031, WT vs. HOM *P* < 0.00001, HET vs. HOM *P* = 0.00057), male (1-way ANOVA, *F*(2,24) = 101.20, *P* = 2.02 × 10^–12^; Tukey’s post hoc test, WT vs. HET *P* = 0.00057, WT vs. HOM *P* < 0.00001, HET vs. HOM *P* < 0.00001)], and (**D**) PKCα [female (1-way ANOVA, *F*(2,25) = 0.76, *P* = 0.48), male (1-way ANOVA, *F*(2,26) = 4.4, *P* = 0.022; Tukey’s post hoc test, WT vs. HET *P* = 0.82, WT vs. HOM *P* = 0.023, HET vs. HOM *P* = 0.081)]. Fluorescence immunohistochemistry was performed on fixed sagittal brain sections from all genotypes (WT, HET, HOM) and sexes (*n* = 3–6 mice). (**B**) Representative images show intensity and localization of PKCγ in whole cerebellum (scale bars: 1 mm, respective color scale indicates relative intensity). Since staining is multiplexed, and representative images are shown from the same sections as Figure 3 but in different channels (see Supplemental Figure 4 for examples of independent and merged images). (**C**) Quantification of the PCL and ML (*n* = 3–6 mice) measured the intensity of localized PKCγ [female (1-way ANOVA, *F*(2,10) = 11, *P* = 0.0033; Tukey’s post hoc test, WT vs. HET *P* = 0.18, WT vs. HOM *P* = 0.0025, HET vs. HOM *P* = 0.036), male (1-way ANOVA, *F*(2,12) = 20, *P* = 0.00015; Tukey’s post hoc test, WT vs. HET *P* = 0.044, WT vs. HOM *P* = 0.00010, HET vs. HOM *P* = 0.0092)]. (**E**) Representative images show intensity and localization of PKCα in whole cerebellum (scale bars: 1 mm, respective color scale indicates relative intensity). (**F**) Quantification of the PCL and ML (*n* = 3–6 mice) measured the intensity of localized PKCα. Bar graphs show mean ± SEM normalized to WT. Significance was determined by 1-way ANOVA with Tukey’s post hoc test. *P < 0.05; **P < 0.01; ***P < 0.001; ***P < 0.0001.

PKCγ protein expression has been reported to be reduced in SCA14 in both humans and mouse models (2, 33, 49, 52). To determine whether ΔF48 PKCγ affected PKC protein expression, immunofluorescent staining of cerebellar sagittal slices and Western blot analysis of whole cerebellar homogenate was performed on all genotypes and sexes for PKCγ and PKCα, another conventional PKC highly expressed in Purkinje cells (36, 50, 51). Western blot analysis identified a ΔF48 PKCγ gene dose–dependent decrease in total PKCγ in the cerebellum. Analysis of HET mice showed a decrease in PKCγ of 41% in females and 27% in males, and HOM mice had PKCγ levels that were severely reduced by approximately 90% in both sexes compared with WT. HOM mice also had significantly lower PKCγ levels compared with HET mice in both sexes (83% lower in females and 87% lower in males) (Figure 4A). This loss in PKCγ was observed throughout all brain regions that express PKCγ, as observed in stained sagittal slices of whole brain (Supplemental Figure 3). To determine where this reduction occurred specifically in the cerebellum, immunofluorescent staining was performed for PKCγ and Calbindin D28k. As expected, PKCγ was localized to Purkinje cells (Supplemental Figure 4) and a similar reduction in PKCγ levels was observed in SCA14 mice of both sexes (Figure 4B). Quantification of the mean intensity of the PKCγ signal in the PCL and ML region revealed an approximately 25% decrease in PKCγ in HET mice for both sexes, and HOM mice displayed a decrease of 69% in females and 62% in males compared with WT (Figure 4C). This reduction in steady-state protein was not a result of altered transcription, as RT-qPCR revealed no significant differences in PKCγ RNA expression in WT versus HOM mice (Supplemental Figure 5). Levels of PKCα were modestly reduced in the SCA14 mice. Western blot analysis of the cerebellum indicated that PKCα levels trended toward a decrease in SCA14 female mice and was significantly reduced by 26% in HOM male mice compared with WT male mice (Figure 4D). Slices stained for PKCα (Figure 4E and Supplemental Figure 4), with quantification of PKCα intensity in the PCL and ML, had no significant changes in PKCα levels (Figure 4F). These results indicate that PKCγ is gene dose–dependently reduced in SCA14 mice, due to the increased steady-state turnover rate previously identified for ΔF48 PKCγ (Figure 1), and that PKCα is not compensating for a loss of PKCγ protein in the Purkinje cells. Note that the modest decrease in PKCα is consistent with ΔF48 PKCγ, which is mostly unphosphorylated and in a more open conformation, dimerizing with other PKC isozymes, impairing their processing phosphorylations and enhancing quality control degradation (57).

### Total PKC substrate phosphorylation and other PKC isoforms are unaltered in SCA14 mice.

**Figure 5:**
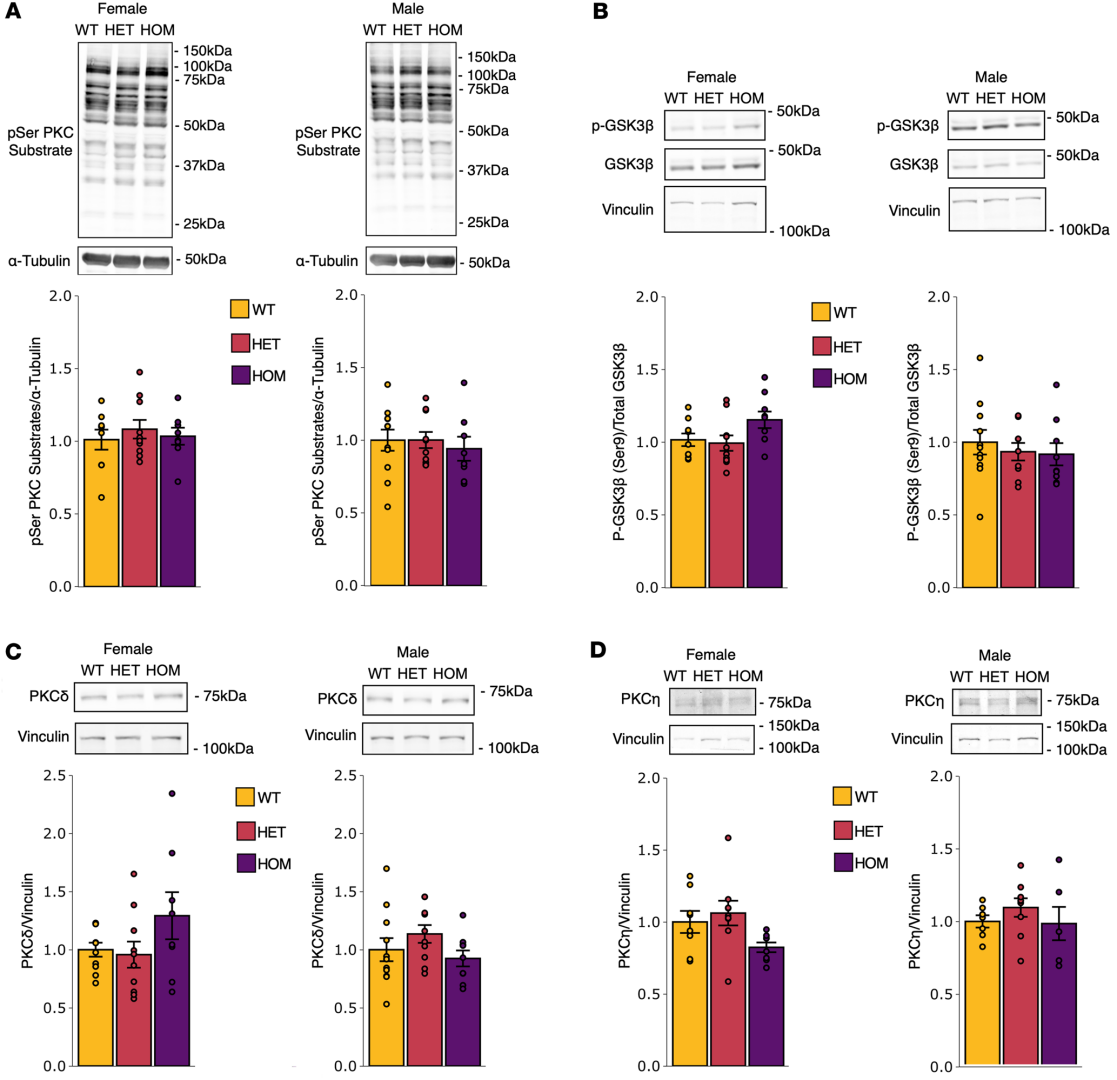
SCA14 mice have equivalent PKC substrate phosphorylation and PKC isozyme levels to those of WT mice. Western blot analysis was performed on whole cerebellar homogenate from all genotypes (WT, yellow; HET, red; HOM, purple) and sexes (*n* = 6–11 mice per group) to assess p-Ser PKC substrate phosphorylation, GSK3β (Ser^9^) phosphorylation, and PKCδ and PKCη expression. Western blot analysis for (**A**) p-Ser PKC substrate phosphorylation sites, (**B**) phosphorylated over total GSK3β, (**C**) PKCδ, and (**D**) PKCη identified no significant differences between genotype or sex. Western blot data are normalized to WT. Bar graphs represent mean ± SEM. Significance was determined by 1-way ANOVA with Tukey’s post hoc test.

Although SCA14 mice have significantly reduced levels of PKCγ protein, we reasoned that the enhanced basal activity of ΔF48 PKCγ could still drive aberrant phosphorylation. To determine whether the observed loss of PKCγ in the cerebellum of SCA14 mice affected overall PKC signaling, we measured total PKC substrate phosphorylation using Western blot analysis of cerebellar homogenate from all genotypes and sexes. No significant differences in substrate phosphorylation were identified despite reduced levels of PKCγ and PKCα in SCA14 mice compared with WT in either sex (Figure 5A). We also assessed phosphorylation of glycogen synthase kinase-3β (GSK3β), which is a protein known to have increased phosphorylation with other overactive, ataxia-associated mutations in PKCγ (31) and contains a bona fide PKC consensus RxxS site on serine 9 (Ser^9^) (31, 58). No significant differences were identified in phosphorylation of GSK3β (Ser^9^) in SCA14 mice in either sex (Figure 5B). To address whether other PKC isoforms may affect total PKC substrate phosphorylation and compensate for the reduction in PKCγ protein, we measured the expression of other PKC isoforms known to be present in cerebellar Purkinje cells, PKCδ and PKCη (36, 50, 51). Western blot analysis revealed no significant differences in the levels of PKCδ (Figure 5C) or PKCη (Figure 5D) between SCA14 genotypes compared with WT in either sex. Thus, although PKCγ is dramatically reduced in the cerebellum of SCA14 mice, its enhanced leaky basal activity results in no overall change in PKC substrate phosphorylation, including phosphorylation of the previously identified site on GSK3β that has been shown to increase in ataxia models of overactive mutant PKCγ (31, 58).

### SCA14 mice have an altered phosphoproteome.

**Figure 6:**
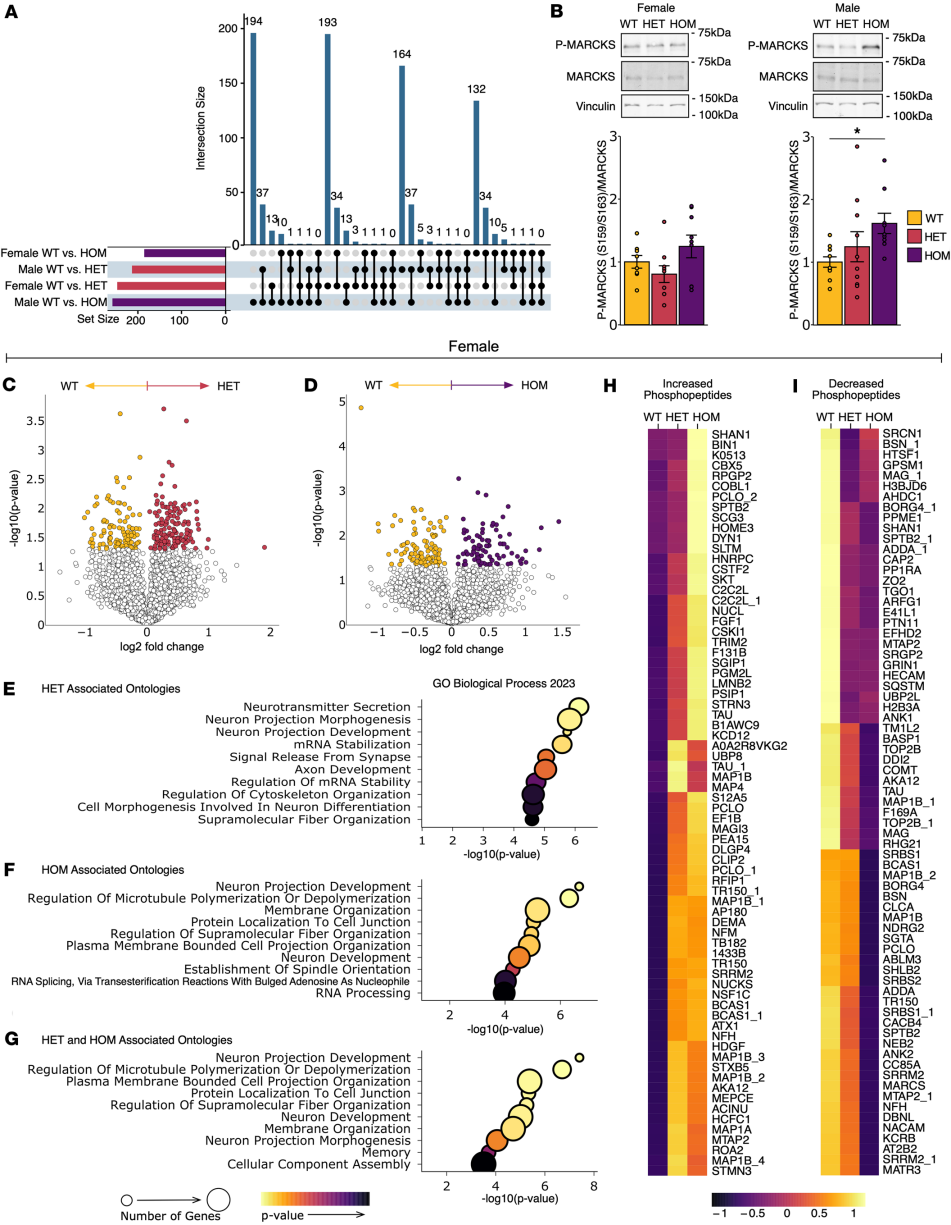
ΔF48 PKCγ rewires the cerebellar phosphoproteome in SCA14 mice. Phosphoproteomic analysis was carried out on protein extracted from whole cerebellar homogenate from all genotypes (WT, yellow; HET, red; HOM, purple) and sexes (*n* = 3 mice per group). (**A**) An upset plot summarizes the quantity of significantly different phosphopeptides and shared phosphopeptides identified between groups (*P* < 0.05). (**B**) Western blot analysis was performed on whole cerebellar homogenate (*n* = 8–10 mice per group) to assess MARCKS phosphorylation (Ser^159^/Ser^163^) [female (1-way ANOVA, *F*(2,23) = 2.4, *P* = 0.11; Tukey’s post hoc test, WT vs. HET *P* = 0.62, WT vs. HOM *P* = 0.48, HET vs. HOM *P* = 0.094), male (1-way ANOVA, *F*(2,26) = 3.1, *P* = 0.063; Tukey’s post hoc test, WT vs. HET *P* = 0.58, WT vs. HOM *P* = 0.051, HET vs. HOM *P* = 0.31)]. Bar graphs represent mean ± SEM. Significance determined by 1-way ANOVA with Tukey’s post hoc test. Volcano plots show log-transformed *P* values versus the log-transformed fold change quantified per change in phosphopeptide abundance in WT compared to (**C**) HET and (**D**) HOM in female mice. Color represents phosphopeptides with *P* value < 0.05. Dot plots indicate the top 10 most significantly enriched for gene ontologies from the differentially abundant phosphopeptides identified in (**E**) WT vs. HET, (**F**) WT vs. HOM, and (**G**) similarly altered phosphopeptides (defined as phosphopeptides significantly altered in HOM vs. WT with a corresponding trend, or significant change, in HET vs. WT) in female mice. Similarly altered phosphopeptides are shown in heatmaps by those that (**H**) increase or (**I**) decrease with increasing ΔF48 alleles in females. Dot plot color scale indicates *P* value, and dot size indicates number of genes per ontology (*P* < 0.05). Heatmap color scale indicates normalized phosphopeptides intensity, with values centered and scaled by row. Protein names accompanied by a number designate multiple phosphorylation sites for the same protein.

To identify the effects of the aberrant ΔF48 PKCγ signaling in the cerebellum, a phosphoproteomic analysis was carried out on protein extracted from whole cerebellar homogenate from all genotypes and sexes. A total of 5216 quantifiable proteins and 7829 quantifiable phosphopeptides were detected. Phosphopeptide intensity was normalized to the corresponding protein intensity. Protein intensity was determined in a parallel proteome analysis (Supplemental Figure 6). Significant differences in phosphopeptide abundance (*P* < 0.05, as determined by 2-sample *t* test) were compared between genotypes in female and male mice. An upset plot displays the significant differentially abundant phosphopeptides found between each SCA14 genotype compared with WT (sets: WT vs. HET and WT vs. HOM) in male and female mice (Figure 6A). Male HOM mice had the greatest number of differentially abundant phosphopeptides and the greatest number not shared with any other sets (set size: Female: WT vs. HET = 246, HOM = 184; Male: WT vs. HET = 212, WT vs. HOM = 257). The intersections indicate that relatively few phosphopeptides were shared between sets, with the male HET and male HOM having the most in common (37 phosphopeptides), followed by female HET and female HOM (34 phosphopeptides) (Figure 6A). No differentially abundant phosphopeptides were found in common between all sets, suggesting that ΔF48 PKCγ rewires the phosphoproteome specifically according to sex.

To validate sex-specific changes, Western blot analysis was performed on cerebellum from mice of all genotypes and sexes for phosphorylated (Ser^159^/Ser^163^) and total myristoylated alanine-rich C-kinase substrate (MARCKS), a bona fide PKC substrate. A significant increase in phosphorylation of MARCKS was observed in HOM males, but not females, compared with WT (Figure 6B). A similar increase was observed in the phosphoproteomic analysis for the same phosphorylation site (Ser^163^) in SCA14 males. Thus, the ΔF48 mutation in PKCγ affects the phosphorylation of diverse substrates, including the well-characterized MARCKS.

**Figure 7:**
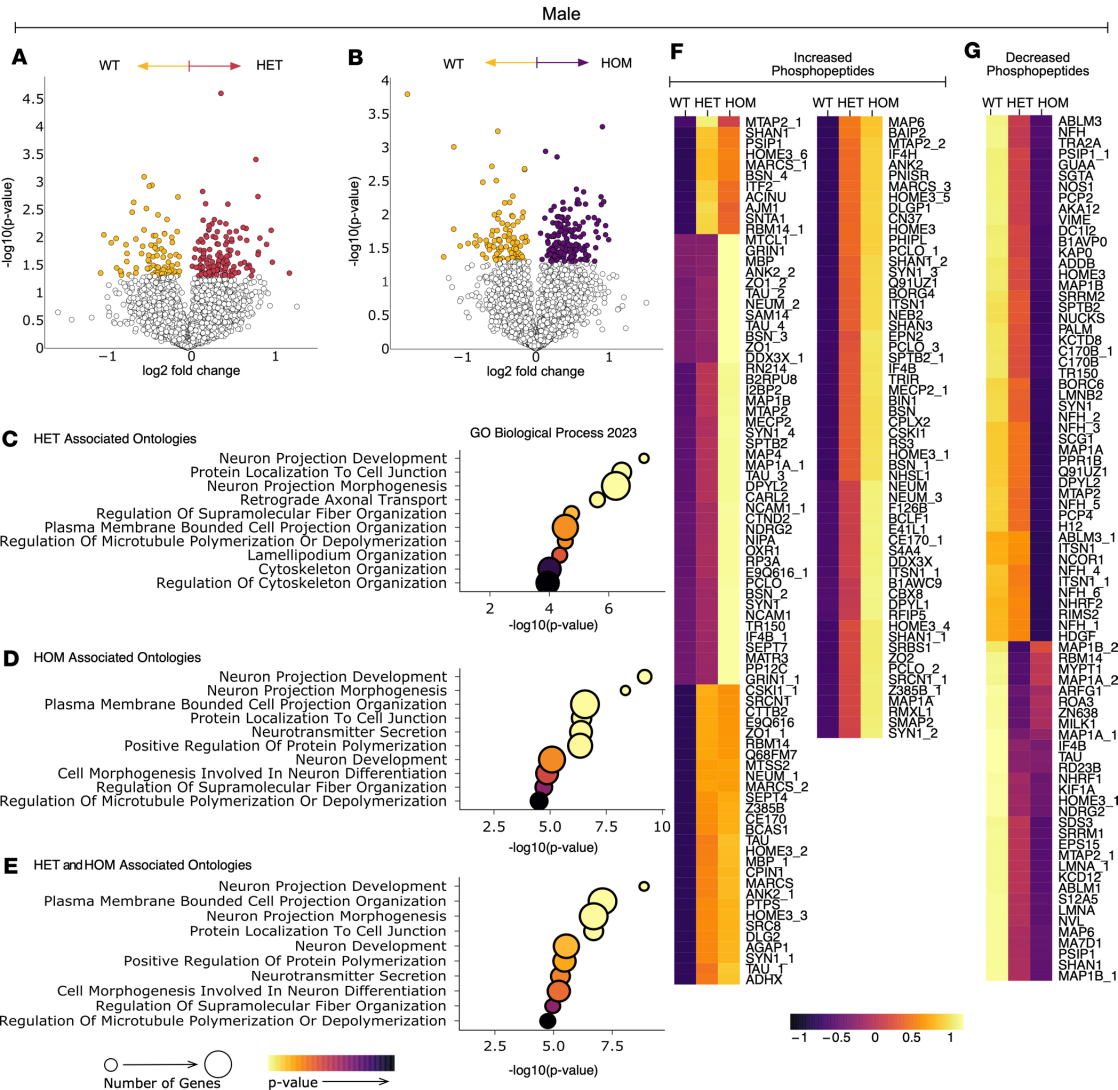
ΔF48 PKCγ rewires the cerebellar phosphoproteome in SCA14 male mice. Phosphoproteomic analysis was carried out on protein extracted from whole cerebellar homogenate from all genotypes (WT, yellow; HET, red; HOM, purple) and sexes (*n* = 3 mice per group). In male mice, volcano plots show change in phosphopeptide abundance between WT and (**A**) HET and (**B**) HOM. Color represents phosphopeptides with *P* value < 0.05. Dot plots indicate the top 10 most significantly enriched for gene ontologies from the differentially abundant phosphopeptides identified in (**C**) WT vs. HET, (**D**) WT vs. HOM, and (**E**) similarly altered phosphopeptides (defined as phosphopeptides significantly altered in HOM vs. WT with a corresponding trend, or significant change, in HET vs. WT) in males. Color scale indicates *P* value, and dot size indicates number of genes per ontology (*P* < 0.05). Similarly altered phosphopeptides are shown in heatmaps by those that (**F**) increase or (**G**) decrease with increasing ΔF48 alleles in males. Dot plot color scale indicates *P* value, dot size indicates number of genes per ontology (*P* < 0.05). Heatmap color scale indicates normalized phosphopeptide intensity, with values centered and scaled by row. Protein names accompanied by a number designate multiple phosphorylation sites for the same protein.

To better understand signaling pathways and cellular functions differentially altered by the ΔF48 PKCγ variant, significant differences in phosphopeptide abundance with SCA14 genotypes were compared in each sex separately. In female mice, volcano plots indicate phosphopeptides that were significantly increased (more abundant in the SCA14 genotype) or decreased (more abundant in WT) in HET (Figure 6C) and HOM (Figure 6D) female mice. Corresponding gene ontology analysis was performed for significantly different phosphopeptides found in HET and HOM compared with WT females for biological processes (Figure 6, E and F), cellular components (Supplemental Figure 7, A and B), and molecular functions (Supplemental Figure 7, C and D). The top 10 most significantly enriched for ontologies are shown (*P* < 0.05), as assessed using EnrichR (59–61). Gene ontology analysis was also performed on significantly different phosphopeptides associated with both HET and HOM female mice. These similarly associated phosphopeptides were determined as phosphopeptides that were significantly altered in the HOM versus WT comparison and had a corresponding trend or significant change in the same direction in the HET versus WT comparison (biological processes, Figure 6G; cellular components, Supplemental Figure 7E; molecular functions Supplemental Figure 7F). These similarly altered phosphopeptides were displayed in heatmaps as those that were increased (Figure 6H) or decreased (Figure 6I) in the SCA14 genotypes compared with WT in females (proteins of the phosphopeptides are labeled on heatmaps). As with the female mice, volcano plots (Figure 7, A and B), gene ontologies (Figure 7, C–E, and Supplemental Figure 7, G–L), and heatmaps (Figure 7, F and G) are displayed using the same analysis for the male mice. Concerning the similarly associated phosphopeptides, 78% of all the differentially abundant phosphopeptides in HOM female mice were similarly associated in HET female mice, and 85% of all the differentially abundant phosphopeptides in HOM male mice were similarly associated in HET male mice. This suggests that both SCA14 genotypes have changes in the same phosphorylation processes, but these are more pronounced in HOM mice; however, male mice had more similarly associated phosphopeptides that increased than females, suggesting increased basal signaling of ΔF48 PKCγ. For both sexes, ontologies were centered on neuronal and cytoskeletal processes. Although ontologies were conserved, the signaling mechanisms involved may be different, as phosphopeptides were not highly conserved between comparisons.

**Figure 8:**
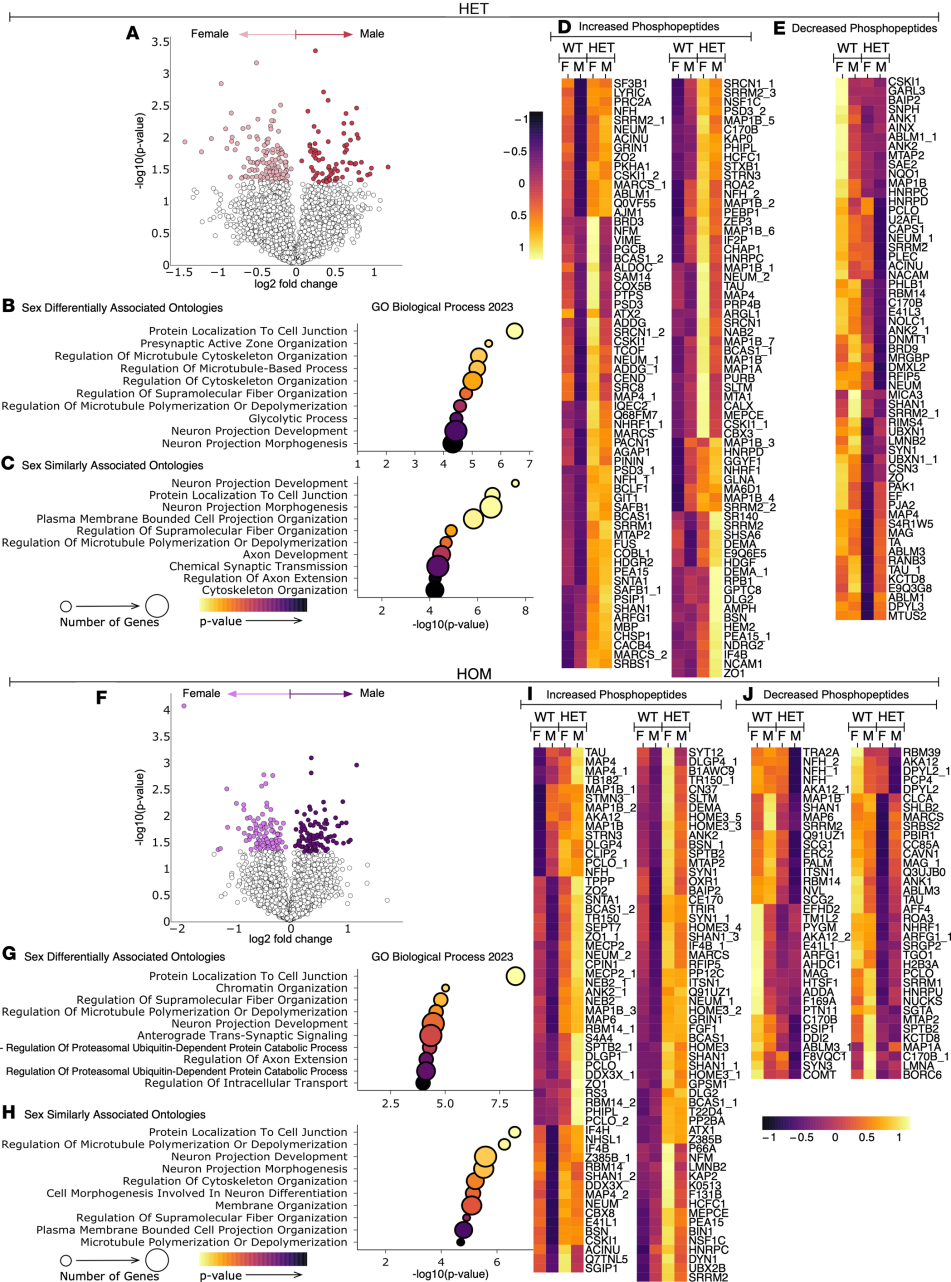
Sex differences in the SCA14 mouse cerebellar phosphoproteome. Phosphoproteomic analysis was carried out as in Figure 5. (**A**) Volcano plots show change in phosphopeptide abundance between male HET and female HET mice. Color represents phosphopeptides with *P* value < 0.05. Dot plots indicate the top 10 most significantly enriched for gene ontologies from the differentially abundant phosphopeptides identified in (**B**) male HET vs. female HET mice, and (**C**) similarly altered phosphopeptides in HET mice (defined as phosphopeptides with the same trend in males and females with a significant difference in intensity between WT vs. HET in at least one sex). Similarly altered phosphopeptides are shown in heatmaps separated by those that (**D**) increase or (**E**) decrease in HET mice compared with WT. (**F**) Volcano plots show change in phosphopeptide abundance between male HOM and female HOM mice. Color represents phosphopeptides with *P* value < 0.05. Dot plots indicate the top 10 most significantly enriched for gene ontologies from the differentially abundant phosphopeptides identified in (**G**) male HOM vs. female HOM mice, and (**H**) similarly altered phosphopeptides in HOM mice (defined as phosphopeptides with the same trend in males and females with a significant difference in intensity between WT vs. HOM in at least one sex). Similarly altered phosphopeptides are shown in heatmaps separated by those that (**I**) increase or (**J**) decrease in HOM mice compared with WT. Dot plot color scale indicates *P* value, and dot size indicates number of genes per ontology (*P* < 0.05). Heatmap color scale indicates normalized phosphopeptides intensity, with values centered and scaled by row. Protein names accompanied by a number designate multiple phosphorylation sites for the same protein.

To further explore sex differences in the phosphoproteome, we compared SCA14 genotypes between male and female mice. In HET mice, volcano plots identified differentially abundant phosphopeptides that were significantly increased (more abundant in male HET) or decreased (more abundant in female HET) (Figure 8A). Corresponding gene ontology analysis was performed for differentially abundant phosphopeptides found in HET male compared with HET female mice for biological processes (Figure 8B), cellular components (Supplemental Figure 7M), and molecular functions (Supplemental Figure 7N). The top 10 most significantly enriched for ontologies are shown (*P* < 0.05). Gene ontology analysis was performed for phosphopeptides that were associated in both sexes. Phosphopeptides that were similarly associated with sex were designated as phosphopeptides that had the same directionality of change in HET versus WT in both sexes and were significantly different in at least one sex (biological processes, Figure 8C; cellular components, Supplemental Figure 7O; molecular functions, Supplemental Figure 7P). These similarly sex-associated phosphopeptides are displayed as heatmaps, with increasing phosphopeptides (Figure 8D) being more prevalent than decreasing phosphopeptides (Figure 8E). As with the HET mice, volcano plots (Figure 8F), gene ontologies (Figure 8, G and H, and Supplemental Figure 7, Q–T), and heatmaps (Figure 8, I and J) are displayed for HOM male mice compared to HOM females. As in HET mice, similarly sex-associated phosphopeptides are displayed as heatmaps, with increasing phosphopeptides (Figure 8I) being more prevalent than decreasing phosphopeptides (Figure 8J). Of the differentially abundant phosphopeptides in HET mice, 41% were similarly altered in both sexes, and 43% were similarly altered in both sexes in HOM mice, suggesting that the majority of altered phosphopeptides in each genotype are not commonly altered in both sexes. Ontologies for these comparisons were centered on neuronal and cytoskeletal processes for both differentially and similarly altered phosphopeptides. Notably, many significant sex-specific changes were associated with synaptic functions. Overlap in ontologies occurred between comparisons; however, altered phosphosites remained unique between groups, suggesting that similar biological processes were affected, but not necessarily through the same pathway.

### Kinase substrate motif enrichment analysis identifies altered kinome signaling in SCA14.

**Figure 9:**
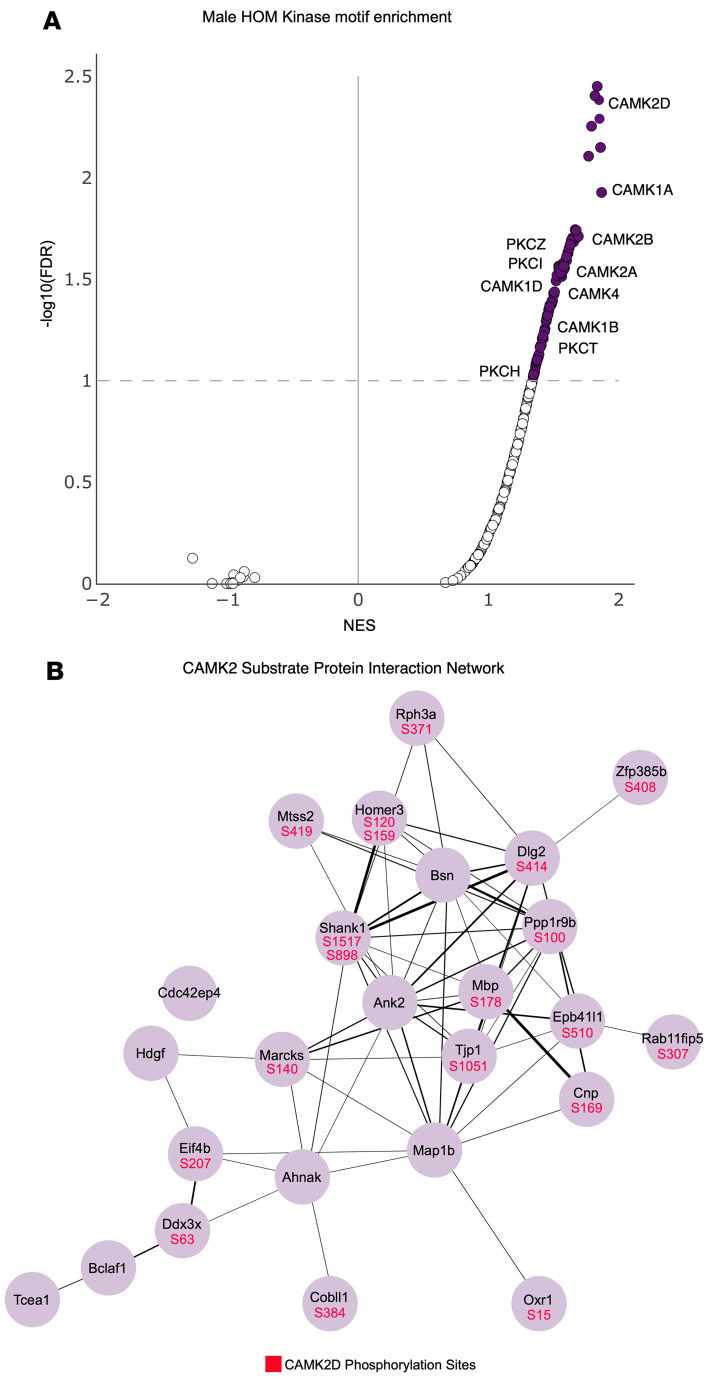
Kinase substrate motif enrichment analysis identified altered kinome signaling in SCA14. Kinase substrate motif enrichment analysis of identified phosphopeptides in WT vs. HOM male cerebellum revealed upregulated substrate motifs corresponding to multiple kinases. (**A**) Volcano plot indicates kinase enrichment in WT vs. HOM male cerebellum. Color indicates significance (FDR < 0.1). (**B**) Protein-protein interaction network generated using STRING shows CAMK2 substrates that were identified from motifs on significantly different phosphopeptides in WT vs. HOM male (*P* < 0.05). Line thickness indicates strength of supporting data (confidence 0.15). Significantly different phosphorylation sites in WT vs. HOM males within the CAMK2D substrates are shown in red within their corresponding protein dot.

To further investigate dysregulated signaling events in SCA14, a kinase motif enrichment analysis was performed on the altered phosphoproteome of the ΔF48 PKCγ cerebella. This analysis compared the sequences of the identified differentially abundant phosphopeptides detected in the ΔF48 PKCγ mice, relative to WT mice, to experimentally determined kinase substrate motifs for the entire human kinome (62, 63). This allowed us to infer the activity of different kinases from changes in kinase substrate motif abundances within our dataset. Using this method, over 100 dysregulated kinases were identified in SCA14 HOM male mice (FDR < 0.1), revealing that aberrant PKCγ activity indirectly or directly altered other kinase activity (Figure 9 and Supplemental Figure 8). Various kinases were predicted to have altered signaling in the SCA14 mice, and each genotype and sex had a unique kinase substrate profile. Only the group with the least severe behavioral phenotypes, the female HET mice, had significantly reduced substrate motifs, while all other phosphoproteome comparisons predicted increased activity of kinases. Surprisingly, PKCγ itself was not identified as having increased activity. Rather, Ca^2+^/calmodulin-dependent kinase 2 (CaMK2) family members had significantly enriched substrates in all phosphoproteome comparisons (Figure 9 and Supplemental Figure 8) and was one of the most significantly predicted kinases to contribute to altered phosphorylation in ΔF48 HOM male mice (Figure 9A). CaMK2 family members are Ser/Thr protein kinases that play a critical role in regulating synapse structure and function through phosphorylation of scaffolds and receptors (64, 65). In this regard, CaMK2 substrates whose phosphorylation was significantly enhanced in the ΔF48 HOM male mice revealed an abundance of synaptic scaffold proteins such as HOME3, HOME4, SHANK1, and DLG2 as well as cytoskeletal proteins such as ANK2 and MARCKS, displayed in a protein interaction network (66) (Figure 9B); these are all known regulators of synapse structure and function (67–69). These data suggest that a major consequence of aberrant PKCγ signaling is increased activity of CaMK2.

### Clinical data indicate that SCA14 symptoms have a later age of onset in female compared with male patients.

**Figure 10:**
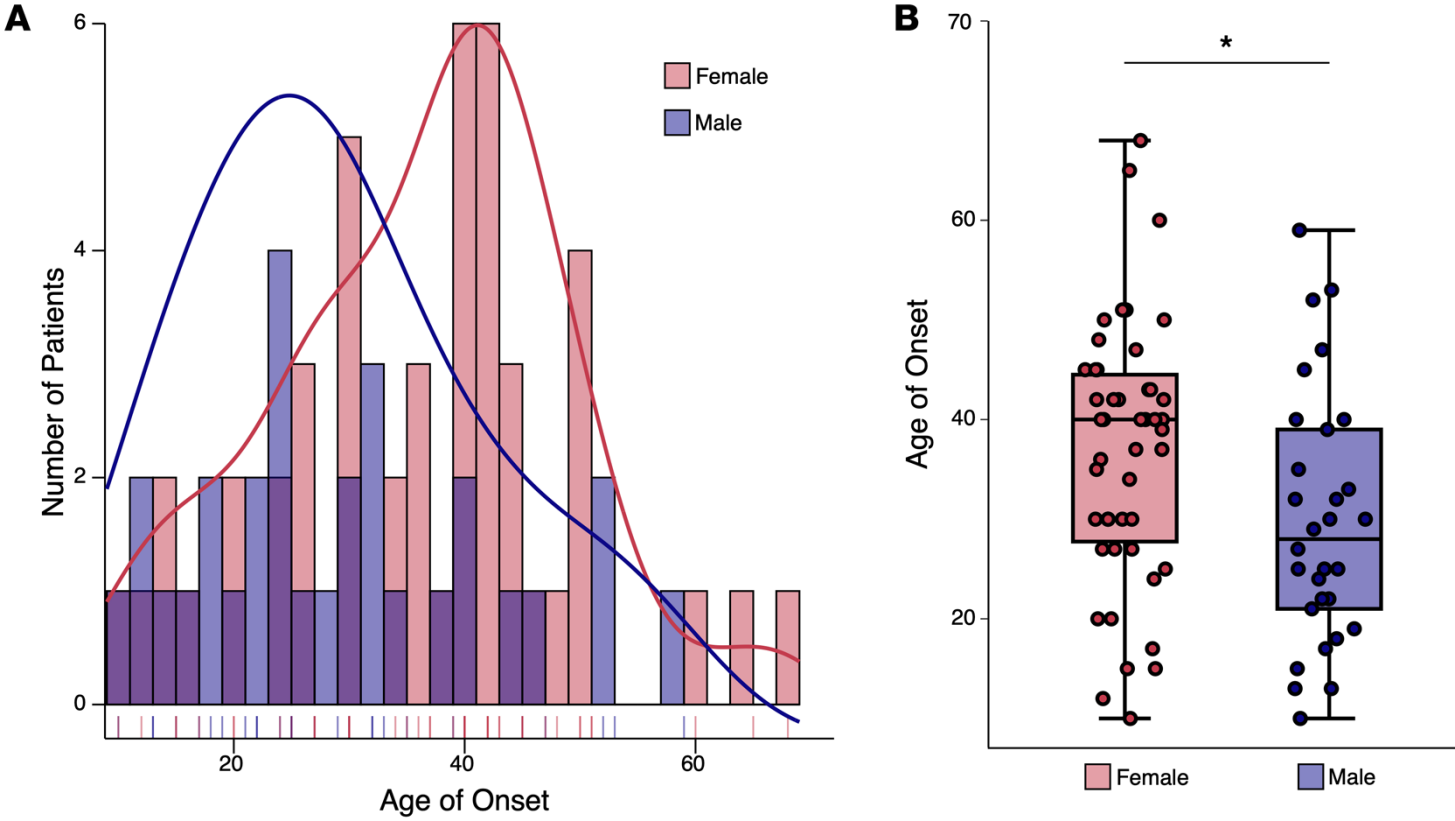
SCA14 has a later age of onset in female patients compared with male patients. Using the OMIM database, data from 77 SCA14 patients were obtained from studies that reported a clinical description that included the age of onset and sex. (**A**) Histogram displays the age of onset reported by SCA14 patients with sex designated by color, and the density curve shows the distribution for female patients (red) and male patients (blue). (**B**) Box-and-whisker plot displays the significant difference in age of onset between female patients (red) and male patients (blue) as determined by 2-sample Student’s *t* test. This plot shows the median (center line), interquartile range (box), and the range of minimum and maximum values (whiskers). *P < 0.05. Age of onset (mean ± SEM): female = 37 ± 2 years, male = 29 ± 2 years.

Having observed sex differences in our SCA14 mice, we investigated human patient data for differences in disease severity in male and female patients with SCA14. Using the Online Mendelian Inheritance in Man (OMIM, #605361) database, we analyzed the available data of 77 patients with SCA14 that had clinical descriptions that included the age of onset and sex of the individuals (5, 13, 70–73). Since ΔF48 PKCγ is a rare mutation that is lacking in patient data, we used data from individuals with a variety of PKCγ mutations causative for SCA14. Interestingly, the reported age of onset of SCA14 symptoms in female patients was significantly higher (37 years old) than in male patients (29 years old) (Figure 10, A and B). Additionally, fewer male patients were identified than female, which could reflect a loss in viability of male humans (30 males and 47 females; Figure 10A). This is consistent with our findings from the mouse model suggesting that female patients may have neuroprotective mechanisms that diminish the onset of symptoms and increase viability.

## Discussion

We have developed an SCA14 mouse model with deletion of residue F48 of PKCγ identified in human patients. This mutation was selected because of the severity of the biochemical defect and resulting severity of the associated symptoms (11, 31). As with other SCA14 mutations, ΔF48 PKCγ has impaired autoinhibition that results in leaky basal activity but is unique in that this mutation breaks communication between the DG sensor (C1B domain) and the autoinhibitory pseudosubstrate (Figure 1). As a result, it is unresponsive to agonist-evoked activation (20, 31, 32), the downstream effects of which are unknown. Thus, the mutation provides a unique tool to examine how leaky basal activity, associated with SCA14 mutations, drives the disease. Behavioral, immunohistochemical, phosphoproteomic, and biochemical analysis revealed motor dysfunction, altered Purkinje cell morphology, a rewired phosphoproteome with notable activation of CaMK2 that affects phosphorylation of neuronal and cytoskeletal processes, and no overall change in PKC substrate phosphorylation despite significantly reduced levels of PKCγ. Strikingly, phenotypes were significantly more severe in males, suggesting a potential neuroprotective mechanism in female SCA14 mice that may also be reflected in SCA14 human patients. Our data reveal that the aberrant signaling of ΔF48 PKCγ is sufficient to drive ataxia-related phenotypes, with male mice displaying greater aberrations.

### SCA14 mice display sex differences that indicate a potential neuroprotective mechanism in female mice.

A major finding in this study was sex differences in our SCA14 mouse model with ΔF48 PKCγ. Little information is available on sex differences in SCA14 human patients or models. This finding highlights an important factor to consider when developing treatment strategies in SCA14 as well as other neurodegenerative diseases associated with PKC activity (74, 75). Notably, our investigation of clinical data identified that the reported age of onset of SCA14 symptoms in female patients was significantly higher than in male patients, and that fewer male patients were identified than female, which could reflect a loss in viability of male humans (5, 13, 70–73). However, a caveat of this finding is that it includes multiple PKCγ-associated SCA14 mutations. Nonetheless, despite a relatively small sample size in the human cohorts, SCA14 appears to affect males more profoundly than females across various SCA14 mutations, warranting attention to sex differences to provide more insight into how prevalent sex differences are in all SCA diseases.

Given the decreased severity of SCA14 symptoms in females, an intriguing possibility is that estrogen, particularly 17-β estradiol (E_2_), may provide a neuroprotective effect. Consistent with this, E_2_ has been shown to be neuroprotective in several diseases, including cerebellar ataxia models. Furthermore, inhibition of PKC has been reported to prevent E_2_ neuroprotection of neuronal cultures (76–84). Notably, estrogen has been reported to increase PKC activity and/or expression in a variety of tissue and cell types, including the brain (85–92). E_2_ induces activation of PLC, leading to the production of DG/Ca^2+^ and thus agonist-evoked activation of PKCγ (90–93). E_2_ has also been shown to modulate Purkinje cell function (41, 94, 95). Since ΔF48 PKCγ is both a gain-of-function (increased basal activity) and loss-of-function (impaired agonist-evoked signaling and loss of dynamics of activity) mutation, perhaps E_2_ can increase cellular DG/Ca^2+^ to enhance signaling by any functional PKC (i.e., WT PKCγ/α) in the cerebellum. This may overcome aberrant ΔF48 PKCγ signaling and allow WT PKCγ to mask the deleterious effects of ΔF48 PKCγ in females. Future research on the role of E_2_ on PKC signaling in SCA14 could explore this hypothesis.

### Increased basal activity of ΔF48 PKCγ compensates for loss of total PKCγ.

Our investigation of PKCγ expression and localization revealed a dramatic gene dose–dependent loss of total PKCγ in both males and female mice. We have previously shown that the cellular turnover of ΔF48 PKCγ is almost an order of magnitude faster than that of WT PKCγ because of inherent instability of the mutant PKCγ (20, 31). Thus, it is not surprising that steady-state levels of ΔF48 PKCγ are low in HET and HOM mice. Aberrant PKC dimerizes with WT enzyme, trapping it in a degradation-sensitive conformation; therefore, levels of WT PKCγ and its paralog PKCα may also be reduced. Unlike PKCγ, PKCα is expressed in other cell types in the cerebellum, which may explain why results from the Western blot and immunofluorescence analysis for PKCα differed. Importantly, the unrestrained activity of ΔF48 PKCγ is sufficient to drive robust substrate phosphorylation. A similar finding was reported for another SCA14 mouse model; a transgenic mouse expressing a pseudosubstrate mutant PKCγ, A24E, displays a 10-fold reduction in PKCγ steady-state levels yet results in a paradoxical increase in cerebellar substrate phosphorylation (33). It is noteworthy that deletion of the PKCγ gene does not produce some characteristic SCA phenotypes (96). This reveals that it is not loss of PKCγ, but the presence of aberrant PKCγ that drives ataxia-associated phenotypes. These findings underscore the importance of unrestrained leaky activity of PKCγ in driving SCA14.

Little is known about PKCγ expression in SCA14 human patients; however, one study of a male patient with the SCA14-associated mutation H101Y in PKCγ reported reduced PKCγ levels in Purkinje cells. Although a loss of Purkinje cells was observed, remaining Purkinje cells with equivalent Calbindin D28k staining to that in control cerebellum also showed reduced PKCγ levels, suggesting the loss of PKCγ was irrelevant to Purkinje cell loss in this patient (2). Kapfhammer and colleagues have previously suggested that it is not Purkinje cell loss, but rather Purkinje cell dysfunction, that drives ataxic phenotypes (49).

### ΔF48 PKCγ causes cerebellar Purkinje cell dysfunction rather than loss in SCA14 mice.

SCA14 pathogenesis can be driven by neurodegeneration of Purkinje cells, or by dysfunction and altered physiological activity of Purkinje cells leading to motor dysfunction (6, 7, 33, 44, 52 , 53, 54). Although neurodegeneration is a hallmark of SCA, Purkinje cell dysfunction and deficits in morphology have been observed in the absence of Purkinje cell death in SCA14 models, and in some cases Purkinje cell dysfunction precedes ataxic phenotypes (33, 44, 45, 49, 52). Additionally, some SCA14 cases in human patients only show mild cerebellar atrophy, suggesting neurodegeneration may not be the only determinant for SCA14 progression (12, 49). We observed subtle changes in Purkinje cell morphology indicative of Purkinje cell dysfunction. These morphological changes may be due to developmental deficits in Purkinje cell arborization, similar to previous studies that showed that enhanced PKC activity compromises dendritic growth (44–49, 97).

A limitation of our study is that it does not address whether ataxic phenotypes of the ΔF48 PKCγ mouse model are developmental or neurodegenerative. In humans, SCA14 onset ranges from early childhood through late adulthood, depending on the magnitude of the biochemical defect of PKCγ (31). PKCγ mutations that cause late SCA14 onset would suggest a progressive degenerative phenotype, but this remains to be established. Some mutations cause symptoms of ataxia to manifest at early ages (11, 31), including ΔF48 PKCγ with one of the strongest biochemical defects (severely impaired autoinhibition) (31). Another mouse model also introduces a mutation that strongly impairs autoinhibition and is associated with early-onset ataxia in humans (A24E PKCγ) (33, 97). Both of these models are well suited to rigorously dissect the role of developmental and degenerative mechanisms in the onset and progression of ataxic phenotypes. We did not observe overt neurodegeneration in the adult ΔF48 PKCγ mice in the present study and have yet to establish a timeline of disease onset and advancement in our mouse model. Developmental deficits may play a role in pathology given the role of PKCγ in Purkinje cell development (44–49, 97) and the observed reduction in HOM male offspring, but this remains an open question. Continued research to uncover the potential effects of aberrant PKCγ signaling during the development and advancement of SCA14 disease progression is paramount.

### Phosphoproteomic analysis indicates altered neuronal and cytoskeletal processes in SCA14 mice unique to genotype and sex.

Phosphoproteomic analysis of the cerebellum of ΔF48 PKCγ mice identified significantly rewired signaling in both male and female mice, with many affected processes related to neuronal signaling. Changes in phosphopeptides associated with neuronal processes likely reflect disrupted signaling associated with Purkinje cell deficits in SCA14 mice. This further implicates Purkinje cell and cerebellum dysfunction in our SCA14 mice. In addition to neuronally related processes, cytoskeleton-associated ontologies were enriched for in the differentially abundant phosphopeptides identified in SCA14 genotypes. PKC is a known regulator of the cytoskeleton and microtubule dynamics; therefore, dysregulation of PKCγ will likely disrupt signaling in cytoskeleton-associated pathways (21). Mice with the SCA14-associated mutation H101Y PKCγ also have changes in the phosphorylation of cytoskeleton-related proteins, including neurofilament proteins that play key roles in axon growth, with aberrations associated with neurodegeneration (31, 98). We have observed similar changes in the phosphorylation of cytoskeleton-associated proteins, including neurofilament proteins (NFH, NFL, and NFM), as well as proteins related to synaptic scaffolding (DLG2, SHAN1, ANK2, HOME3, among others) in our SCA14 mice, suggesting that ΔF48 PKCγ dysregulates signaling pathways involved in the regulation of neuron cytoskeletal structures and functions. Moreover, cytoskeleton dynamics in the brain are critically involved in neuron and synapse formation and maintenance (21), further implicating dysregulated PKCγ signaling and phosphoproteomic rewiring as an underlying cause of Purkinje cell and cerebellum dysfunction. Additionally, some ontologies that were enriched for in multiple comparisons had a limited number of shared differentially abundant phosphopeptides between groups. Although the same ontologies and pathways are altered in SCA14 mice, the underlying mechanism may differ based on sex and genotype, adding further complexity to SCA14.

A surprising finding from our study is that PKC substrate phosphorylation was not particularly enhanced in the ΔF48 PKCγ cerebellum. Rather, CaMK2 was the kinase responsible for the most phosphorylation changes. Other kinases were also predicted to contribute to the phosphoproteomic rewiring observed in SCA14 cerebellum. Thus, aberrant ΔF48 PKCγ results in enhanced activity of the CaMK2 family of kinases. CaMK2 is established as a master regulator of synaptic function, and CaMK2 substrates are often protein scaffolds that coordinate receptors, effectors, and regulators of synaptic functions (99, 100). These scaffolds also bind PKC to coordinate its downstream signaling, and ΔF48 PKCγ may potentially dysregulate CaMK2 signaling on protein scaffolds (99, 100). The CaMK2 motifs identified were found on several substrates related to protein scaffolding, such as DLG2, HOME3, SHAN1, ANK2, among others, in the phosphopeptides differentially expressed in male HOM mice. These scaffold proteins are components of a polymeric network in the postsynaptic density and help coordinate synaptic signaling (101). This is consistent with the ontologies (e.g., cytoskeleton organization) that were enriched for in the differentially abundant phosphopeptides identified in SCA14 genotypes. This kinome analysis further highlights the importance of tightly regulated kinase signaling and the phosphoproteome in Purkinje cell and cerebellum function. Whether the activation of CaMK2 is through a catalytic event from the aberrantly leaky activity of ΔF48 PKCγ or by altered protein interactions resulting from the slightly open conformation of ΔF48 PKCγ remains to be determined. This will be important for understanding whether aberrant PKCγ should be inhibited or degraded as a therapeutic avenue in SCA14.

Aberrant signaling of ΔF48 PKCγ was sufficient to rewire the cerebellar phosphoproteome uniquely by genotype and sex. Notably, male HOM mice showed the greatest number of significantly altered phosphopeptides, supporting our hypothesis that males with SCA14 are affected more severely. Importantly, of the identified HET and HOM similarly associated phosphopeptides in SCA14 male mice, the majority were increased (64%), and male mice had 67 more phosphopeptides increased compared with females. This may suggest a higher degree of overactivity of ΔF48 PKCγ in male mice and more deficits in signaling pathways. This was also reflected in the similarly sex-associated phosphopeptides, where in both HET and HOM mice more phosphopeptides were increased than decreased. Phosphopeptides that were significantly decreased in SCA14 genotypes, despite the overactive ΔF48 PKCγ, likely reflects PKC regulation of phosphatase function to reduce phosphorylation of some proteins (102).

When comparing HET and HOM similarly altered phosphopeptides, the majority of phosphopeptides trended the same within sex, suggesting that HOM mice may have similar, but more severe, changes in the phosphoproteome to those of HET mice. These findings add validity and biological relevance to our SCA14 mouse model, where HOM animals may be used in studies for a more robust and clear readout while still representing autosomal dominant SCA14 in humans. This is reflected in our behavioral and molecular studies, where HOM mice often displayed a robust, significant change while HET mice displayed a trend in the same direction, with the exception that female mice often had confounding results potentially due to neuroprotective mechanisms. Therefore, our SCA14 mouse may be a good representation for continued experimentation, including future research to modulate PKCγ signaling and SCA14 phenotypes. Furthermore, the limited number of individuals identified with ΔF48 PKCγ (11) occludes establishment of typical disease progression or sex differences, warranting a model to study its underlying mechanism. In the event our model does not recapitulate the pathology of ΔF48 PKCγ in humans, our model will still shed light on other SCA14 mutations and other neurodegenerative diseases that present dysregulated PKCγ signaling (74, 75).

In summary, our study identifies sex-specific differences in the biochemical, cellular, and behavioral effects of the ΔF48 PKCγ SCA14 mutation, with males significantly more affected than females. Further studies are needed to decipher the molecular mechanisms driving these differences, with one possible mechanism being protective effects of estrogen. Our data suggest that treatment strategies to reduce aberrant ΔF48 PKCγ may be particularly beneficial in males.

## Methods

See Supplemental Methods for details on the generation of transgenic mice, behavioral testing, real-time qPCR, immunofluorescence, Western blot analysis, phosphoproteomics, and kinase enrichment analysis.

### Sex as a biological variable.

Sex was considered as a biological variable and all tests utilized male and female mice. This study examined male and female mice in all genotypes (WT, HET, HOM). Sex-dimorphic effects are reported for the SCA14 mouse model.

### Statistics.

Statistical comparisons were made using 1-way analysis of variance (ANOVA), Student’s *t* test, or χ^2^ test where indicated. In all analyses, a *P* value of less than 0.05 was considered significant. No age-related differences were observed between the ages of mice tested; therefore, data from age-matched mouse cohorts of different ages were combined if applicable after normalization to the WT group within each age-matched cohort. All groups (sex and genotype) were present in each cohort when tested. Outliers were determined using Grubbs’ test (α = 0.05) where appropriate. All bar plots are shown as mean ± SEM. Analysis, statistics, and graphical representations were prepared in R programming (version 4.3.3; R studio version 2023.12.1+402) (103). The protein interaction network was generated using STRING (version 12) (66).

### Study approval.

All procedures were approved by the Institutional Animal Care and Use Committee at UCSD and were performed in accordance with the NIH *Guide for the Care and Use of Laboratory Animals* (National Academies Press, 2011).

### Data availability.

The mass spectrometry phosphoproteomics and proteomics data have been deposited to the ProteomeXchange Consortium via the Proteomics Identification Database (PRIDE) (104) partner repository with the dataset identifier PXD060630. The whole-genome sequencing data have been deposited to the NCBI Sequence Read Archive (SRA) repository with the accession PRJNA1442142. Data for all figures are available in the Supporting Data Values file.

## Author contributions

ACN and SAW conceived the project, designed experiments, and wrote the manuscript. SAW and CC performed the experiments. YM performed experiments under the supervision of SST. CAP and SRL coordinated and generated the mouse model and provided subject matter expertise. BAH and KJ performed whole-genome sequencing and analysis and provided subject matter expertise. GG analyzed patient data and provided subject matter expertise. MG performed mass spectrometry analysis and provided subject matter expertise. TMYB, JLJ, and LCC provided subject matter expertise and/or analyzed kinase substrate motif enrichment. AJR and SW performed behavioral experiments. AJR provided subject matter expertise. SAW analyzed data for and generated all figures. All authors edited the manuscript.

## Conflict of interest

LCC is a founder and member of the board of directors of Agios Pharmaceuticals and is a founder and receives research support from Petra Pharmaceuticals; is listed as an inventor on a patent (WO2019232403A1, Weill Cornell Medicine) for combination therapy for PI3K-associated disease or disorder, and the identification of therapeutic interventions to improve response to PI3K inhibitors for cancer treatment; is a co-founder and shareholder in Faeth Therapeutics; has equity in and consults for Cell Signaling Technologies, Volastra, Larkspur, and 1 Base Pharmaceuticals; and consults for Loxo-Lilly. TMY is a co-founder of DeStroke. JLJ has received consulting fees from Scorpion Therapeutics and Volastra Therapeutics.

## Funding support

This work is the result of NIH funding, in whole or in part, and is subject to the NIH Public Access Policy. Through acceptance of this federal funding, the NIH has been given a right to make the work publicly available in PubMed Central.

- NIH grant R35 GM122523 (to ACN).
- NIH/NINDS grant R01 NS120725 (to ACN and SST).
- NIH grants R35 CA197588, P01 CA120964, and P01 CA117969 (to LCC).
- NIH grants P30 CA023100 and P30 DK063491 (to the UCSD Transgenic Mouse Core).
- Claudia Adams Barr Program for Cancer Research award (to JLJ).
- NIH Shared Instrument Grant S10 OD026929 (to the UCSD IGM Genomics Center utilizing an Illumina NovaSeq X Plus).

## Supplementary Material

Supplemental data

Unedited blot and gel images

Supporting data values

## Version 1 04/02/2026

In-Press Preview

## Version 2 05/22/2026

Electronic publication
