# Supplementary material for: Sex-specific disruptions in PKCγ signaling in a mouse model of spinocerebellar ataxia type 14
Source: JCI Insight. 2026 Apr 2;11(10):e192155. doi: 10.1172/jci.insight.192155 (PMC13232732; doi:10.1172/jci.insight.192155)
Supplement: Supplemental data [file jciinsight-11-192155-s224.pdf]

Methods

Generation of transgenic mice

CRISPR target sequence design: gRNAs were designed using available online tools such as CRISPR RGEN Tool (http://www.rgenome.net/) and CRISPRDirect (https://crispr.dbcls.jp). gRNAs were prepared via the annealing of crRNA (5' GTCGGTACAGTGACTGCAGA 3') and tracrRNA (Alt-R® CRISPR-Cas9 tracrRNA; cat. No. 1072532) from Integrated DNA Technologies (Coralville, IA, USA). The crRNA and tracrRNA were chemically synthesized, and RNase-Free HPLC purified by Integrated DNA Technologies (Coralville, IA, USA). Single-strand oligonucleotide (ssODN, 5' AGAGCCACAAGTTCACCGCTCGTTTCTTCAAGCAGCCAA**CAT**GCAGTCACTGTACCGACTT CATCTGGTGAGGGAAGCGGGCTAGGGGAG 3') was chemically synthesized and standard desalted by GENEWIZ (South Plainfield, NJ, USA).

Cas9 protein: Cas9 protein (Alt-R S.p. Cas9 Nuclease V3; cat. no. 1081058) was obtained from Integrated DNA Technologies (Coralville, IA, USA)

Mixture preparation for the microinjection: Cas9 protein, the duplex of crRNA and tracrRNA, and ssODN were diluted and mixed in IDTE buffer from Integrated DNA Technologies (Coralville, IA, USA) to a working concentration of 30 ng/µl, 0.6pmol/µl, and 10 ng/µl, respectively. The mixture was incubated at room temperature for 10 minutes.

Embryo recovery, microinjection, and embryo implantation: Pronuclear-stage embryos were recovered from the superovulated 3-5 weeks old C57BL/N females by the intra-peritoneal (IP) injection of pregnant mare serum gonadotropin (PMSG) at 5 units/mouse (cat. no. HOR-272, Prospec, East Brunswick, NJ, USA) followed by human chorionic gonadotropin (hCG, 5 units/mouse, cat. no. C8554, Millipore Sigma, St. Louis, MO, USA) over 46-48 hours interval later and then mated overnight with C57BL/N males. Pronuclear-stage embryos were harvested

16 hours after HCG injection. The ampulla region of oviducts was washed in M2 medium (EmbryoMax M2 medium; cat. no. MR-015-D, Millipore Sigma, St. Louis, MO, USA) and transferred into 0.5 mg/ml of hyaluronidase (cat. no. H4272, Millipore Sigma, St. Louis, MO, USA). The eggs were released by the punch with needle. After 1 minute, the cumulus cells were separated, and the pronuclear-stage embryos were washed in fresh M2 medium three times and incubated in 30µl of M2 medium at 37°C until the microinjection is completed. For microinjection, the pronuclear-stage embryos were transferred to 100 µl of M2 medium in a cover glass and microinjected with 1~2 picoliters of the mixture using an inverted microscope (Eclipse Ti, Nikon, Tokyo, Japan) equipped with a Femtojet express microinjector (Eppendorf, Enfield, CT, USA). Microinjected pronuclear-stage embryos were transferred into the ampulla of pseudo-pregnant females.

Sequencing and Validation: To validate the deletion of F48 in the PKCγ gene and assess off target effects, whole genome sequencing was performed on WT and ΔF48 PKCγ mice. 250 ng of Genomic DNA from each sample was fragmented by Adaptive Focused Acoustics (E220 Focused Ultrasonicator, Covaris, Woburn, Massachusetts) to produce an average fragment size of 500 base pairs (bp). Sequencing libraries were generating using the KAPA Hyper Prep Kit (KAPA Biosystems, Wilmington, MA, USA) following manufacturer's instructions using 4 cycles of amplification. The quality of the library was assessed using High Sensitivity D1000 kit on a 4200 TapeStation instrument (Agilent Technologies, Santa Clara, CA, USA).

Sequencing was performed using the Illumina NovaSeq X Plus Sequencing System (Illumina, San Diego, CA, USA), generating 150 bp paired-end reads to obtain 30x coverage. The sequencing data was run through the DRAGEN Germline Pipeline (DRAGEN v4.4.6, Illumina, San Diego, CA, USA) in order to align to genome mm10 and call variants(1). gVCFs were merged and normalized using bcftools (samtools/bcftools version 1.14) before assessing the variants in the PKCγ as well as off target regions(2). The aligned sequences were visualized using Integrated

Genomic Viewer (IGV version 2.19.6) to confirm incorporation of the mutation with no off-target effects from CRISPR/Cas9-mediated genome editing(3) (**Supplemental Figure 1).**

To additionally control for off-target effects, mice were backcrossed a minimum of three times with new WT mice, and colony maintained with regular incorporation of WT mice in the breeding strategy. All mice were genotyped to determine if they were WT, HET, or HOM for the mutant ΔF48 PKCγ gene.

Genotyping: Tissue obtained from mouse ear clips were digested with tail lysis buffer (100 mM Tris pH8, 20 mM NaCl, 5 mM EDTA, 0.2% SDS, with 0.2 mg/ml Proteinase K) and incubated at 56°C for ~18 hours to digest tissue. Genomic DNA was isolated by isopropanol precipitation and resuspended in H_2O. The PKCγ gene was then amplified by PCR from the purified genomic DNA. PCR amplification was performed using Q5 PCR Master Mix (New England Biolabs, cat. no. M0492) with the forward primer for 5'-AGGTGCTGAGAGCGAAGC-3' and the reverse primer for 5'-GGGTGAAATTGGGAAACTGC-3' (reverse complement: 5'-GCAGTTTCCCAATTTCACCC-3'). PCR was designed to amplify both the WT and mutant form of PKCγ. DNA was subjected to restriction enzyme digestion by NspI (NspI restriction digest site is present only in ΔF48 PKCγ alleles) in CutSmart Buffer (New England Biolabs, cat. no. B6004), and visualized via electrophoresis on a 0.8% agarose gel in Tris-acetate-EDTA buffer with Sybr Safe (Invitrogen, cat. no. S33102) to determine the genotype [WT/WT (WT), WT/ΔF48 (HET), or ΔF48/ΔF48 (HOM)]. The mutant ΔF48 PKCγ gene contains an Nsp1 restriction enzyme cut site while WT does not, therefore, restriction enzyme digest will cut ΔF48 PKCγ producing two smaller bands (300 and 400bp) and not WT PKCγ (700bp). Supplemental Figure 1D-E shows the PKCγ sequence, primer design, Nsp1 restriction digest site, and the DNA gel results obtained from this genotyping strategy.

Behavior testing

For behavioral experiments, mice were acclimated to the procedure room prior to testing. Mice were returned to their home cage at the end of the test session. The arena/equipment was wiped down with 70% ethanol before the start of each run. Experimenter was blind to genotype for all tests performed, and each group (Male WT, Male HET, Male HOM, Female WT, Female HET, Female HOM) contained animals from at least three separate litters. Each test was performed at least twice with a minimum of 24 hours rest between each test session, and data are averaged across trials. Multiple age-matched cohorts were tested, and all groups were represented in each cohort. No age-related differences were observed based on the age of the mice tested, and ages were combined (age-matched=5-10 months). Data from age-matched mouse cohorts of different ages were combined after normalization to the WT group within each age matched cohort, where all groups were present in each cohort when tested. Sample sizes reflect availability of mice per genotype (N=5-16). Data are normalized within cohort to the respective WT group when combined to avoid batch effects.

Rotarod test: To test locomotor coordination and balance, the Rotarod test was performed by placing mice on 3cm rotating drum (Accurotar, Omnitech Electronics, Inc., Columbus, OH) and measuring the latency to fall. The rod started in a stationary state and then began to rotate with a constant acceleration of 10 rpm. Three trials were performed for four days and averaged per animal.

Treadmill walking test: For this test, mice were first trained to walk on the treadmill (Columbus Instruments Exer-3/6, Columbus, OH) in three daily 5 min sessions in which stopping resulted in the mice touching the back of the apparatus and experiencing a mild shock (200 msec pulses of electric current with pulse repetition rate of 3 times per second (3 Hz) and an intensity of 1 mA). One week later, the mice were run at a speed of 10 cm/s for one minute without receiving shock and the percentage of time the mice walked ahead of the bumper/rear of the apparatus was assessed. Behaviors such as sliding, facing backwards, or climbing were subtracted.

Ladder rung test: To assess skilled walking and coordination, mice were first trained to walk across a horizontal ladder with evenly spaced rungs (1cm apart) to reach their home cage, then tested three additional times with the spacing of the rungs randomized (i.e. rungs removed at random, with more rungs removed in each subsequent trial). The number of times the mouse's paw slipped from a ladder rung was counted (from video recordings) and averaged over the three random trials.

Wire hang test: The hanging wire test allows for the assessment of grip strength and motor coordination. A 2 mm wire was securely suspended between two stands at a height of 37 cm. Padding was placed in a cage under the wire to safely catch the mice after they fall. Mice were suspended by the tail and gently lowered until they grasped the wire with their front paws. The tail was then released, and the mice were allowed to hang from and move on the wire freely. The mice were timed until they fell from the wire (maximum 120 seconds per trial). The latency to fall time was measured and averaged over two trials.

Grip strength test: Forelimb grip strength was acquired with a digital Grip Strength Meter (Columbus Instruments Model 0167-004L, Columbus, OH). Each mouse was held so that only the forelimb paws grasped the flat mesh assembly and then pulled back until its grip was broken. The peak force reached was averaged across three consecutive trials.

Real-time qPCR

Cerebellum samples from WT, HET, and HOM male and female mice at 6 and 8 months old were dissected, flash frozen in liquid nitrogen, and cryo-pulverized on dry ice using a stainless-steel mortar and pestle. Crushed cerebellum was transported to a 1.5 ml tube. RNA was extracted using TRI reagent (Sigma-Aldrich, cat. no. T9424). The TRI reagent protocol was followed for RNA isolation or extraction per manufacturers recommendation and resuspended in RNase free water (TRI reagent; Sigma-Aldrich, cat. no. T9424). RNA was assessed with a Nanodrop spectrophotometer (Thermo Fisher Scientific). Next, cDNA was synthesized using SuperScript III

first-strand synthesis system for RT-qPCR with 500 ng of RNA input (Invitrogen, cat. no. 108080-051). A control sample lacking reverse transcriptase was included for each sample to assess gDNA contamination. RT-qPCR was run using TB Green premix Ex Taq II Kit (TAKARA, cat. no. RR820A) on QuantStudios III PCR (Applied Biosystems). Standard curves were used to assess efficiency and detection limits of primer sets and determine the appropriate quantity of RNA before testing samples (PKCγ: forward primer 5'-ACCAGGGCATCATCTACAGG-3' and reverse primer 5'-CTTCCTCATCTTCCCCATCA-3'; GAPDH: forward primer 5'-AGGTCGGTGTGAACGGATTTG-3' and reverse primer 5'-TGTAGACCATGTAGTTGAGGTCA-3'). Samples were run in triplicate and the ΔΔCt method was used to analyze the data. No significant differences were observed between male or female WT samples. Experimenter was blind to genotype and sex where applicable. Graphs represent mean±S.E.M. normalized to the respective WT control group, N=5-9 mice.

Immunofluorescence

Tissue preparation: Mice were anesthetized (isoflurane) and perfused with cold PBS followed by freshly prepared 4% paraformaldehyde (PFA) (age-matched=5-10 months). Mice were decapitated and skulls were immersion fixed in 4% PFA overnight at 4°C, followed by brain dissection and an additional immersion fixation in 4% PFA at 4°C for 1 hour. Brains were then cryoprotected by immersing them in 30% sucrose (w/v) in PBS at 4°C until they sink. Brains were then placed in cryomolds (Polysciences Inc., cat. no. 18646D-1) with OCT (VWR, cat. no. 25608-930). Molds were placed into a dry ice/ethanol bath until frozen. Brains were demolded and stored at -80°C until sectioning by the UCSD histology core facility. Brains were sectioned sagittaly into 40 µm sections using a cryostat and kept at -20°C floating in cryoprotectant media.

Immunostaining: The sections were treated in a 24-well plate on a shaker. They were first treated with 0.3% Triton X-100 in PBS for 10 minutes to remove lipids, then treated with a blocking solution (3% donkey serum, 1% BSA, 1% Fish Gelatin, 0.1% Triton X-100, 50 mM

Glycine in PBS) for 1 hour in room temperature. Afterward, they were incubated with antibodies as listed below, sequentially, in cold room overnight. The first antibody is rabbit anti-PKCγ (GeneTex, cat. no. GTX107639), the second antibody is mouse anti-Calbindin D28k (Swant D28k, cat. no. CB300), the third antibodies are a combination of AF568 Donkey anti-Rabbit IgG (Invitrogen, A10042) plus AF680 Donkey anti-mouse IgG (Invitrogen, cat. no. A10038) plus Cy2 Donkey anti-Guinea pig IgG (Jackson immune research, cat. no. 706-225-148**),** and the last antibody is AF790 Rabbit anti-PKC alpha (Santa Cruz Bio, cat. no. sc-8393). Finally, the samples were incubated with freshly prepared 100 ng/ml DAPI in PBS for 5 minutes before mounted on glass with a water-based medium (SouthernBiotech, cat. no. 0100-20). Confocal images were obtained with a Nikon eclipse Ti2 microscope in UCSD Nikon Image Center with a 20x lens with a numerical aperture of 0.75. Z-stacks were taken through the whole cerebellar section at 2 μm intervals, and the entire cerebellum was tile scanned and stitched together. Microscope settings were kept the same for all slices during image acquisition.

Image analysis: Image analysis was performed using FIJI (ImageJ version 2.14.0/1.5f)(4) to quantify the PCL and ML of images. The cerebellar image z-stacks were average projected. Next, a Region of Interest (ROI) was created for the PCL and ML, using a mask for all areas expressing Calbindin D28k, created by auto-thresholding Calbindin D28k images with manual adjustment to the ROI if needed to only select for the PCL and ML. Mean fluorescent intensity was measured within this ROI to obtain relative levels of immunofluorescent staining intensity for each protein interrogated. Background was also measured and subtracted per sample. Mean fluorescent intensity was normalized to WT controls within imaging sessions. The ROI was used to unbiasedly obtain the thickness of the PCL and ML by using the Local Thickness plugin(5). To obtain the linear density of Purkinje cells, the PCL was then traced through all lobules, and Analyze Particle was used to unbiasedly and automatically count the Purkinje somas along the traced selection. Purkinje cell counts were then divided by the selection distance to normalize for variations in distance. Experimenter was blind to genotype and sex for all steps in the

immunostaining process when possible. Data is represented as mean±S.E.M. and intensity values were normalized to the respective WT control group, N=3-6 mice. No significant differences were observed between male or female WT samples. All image analysis software settings were kept the same between samples and only raw images were analyzed. Brightness and contrast were adjusted in the representative images and all represented images were adjusted the same.

Western blot analysis

Cerebellum samples from WT, HET, and HOM male and female mice at 6 and 8 months old were dissected, flash frozen in liquid nitrogen, and cryo-pulverized on dry ice using a stainless-steel mortar and pestle. Crushed cerebellum was transported to a 1.5 ml tube and lysed in Tris-lysis buffer (1 M Tris pH 7.4, 10 mM Sodium Pyrophosphate, 50 mM sodium fluoride, 5 mM EDTA, 1% Triton X-100) and briefly sonicated. Lysis buffer was supplemented with 1 mM PMSF, 50 μg/ml leupeptin, 1 mM Na_3VO_4, 2 mM benzamidine, 1 μM microcystin, and 1 mM DTT added immediately prior to lysis. Protein concentration was quantified by BCA (cat. no. 23227, Pierce). Samples were boiled in sample buffer (250 mM Tris HCl, 8% (w/v) SDS, 40% (v/v) glycerol, 80 μg/ml bromophenol blue, and 2.86 M β-mercaptoethanol) for 5 minutes at 95°C. SDS-PAGE was performed on protein samples (10-20 μg) using 10% acrylamide gels (bis/acrylamide solution, cat. no. 161-0156, BioRad) with protein ladder (cat. no. 161-0394, BioRad). Gels were transferred to 0.45 μm nitrocellulose membranes (cat. no. 1620115, BioRad) by a wet transfer method at 4°C for 1 hour at 100 V in transfer buffer (200 mM Glycine, 25 mM Tris Base, 20% Methanol). Membranes were blocked in 5% (w/v) nonfat dry milk in phosphate buffer saline with tween-20 (PBS-T) for 1 hour at room temperature, then incubated in primary antibodies diluted in 1% BSA in PBS-T overnight at 4°C. Primary antibodies were against the following: PKCα (1/1000), pSer PKC substrate (1/1000), PKCγ (1/1000), α-Tubulin (1/10,000), Calbindin D28k (1/1,000), PKCδ (1/1000), Vinculin (1/1000), phospho-GSK3 α/β(Ser21/Ser9) (1/1000), total GSK3 α/β (1/1000),

phospho-MARCKS (Ser159/Ser163) (1/1000), total MARCKS (1/10,000), PKCη (1/1000), or GAPDH (1/5000). Membranes were washed for 5 minutes three times in PBS-T and incubated with goat anti-rabbit 800 nm and or goat anti-mouse 700 nm secondary antibodies (1/10,000 in 1% BSA in PBS-T) for 1 hour at room temperature, then imaged using Azure Biosystems Sapphire FL Biomolecular Imager and raw images were quantified with FIJI (ImageJ version 2.14.0/1.5f). No significant differences were observed between male or female WT samples. Experimenter was blind to genotype and sex where applicable. Graphs represent mean±S.E.M. normalized to the respective WT control group within gel, N=6-11 mice.

Antibodies

Antibodies used are listed with the company from which they were purchased and catalog number: PKCα (BD Transduction, cat. no. 610108), pSer PKC substrate (Cell Signaling, cat. no. 2261, lot 26), PKCγ (Santa Cruz, cat. no. C-19; or GTX, cat. no. 107639), PKCα (BD Transduction, cat. no. 610108; or Santa Cruz Bio, cat. no. sc-8393), α-Tubulin (Sigma Aldrich, cat. no. T6074), Calbindin D28k (Swant, cat. no. 300), PKCδ (BD Transduction, cat. no. 610397), phospho-GSK3 α/β(Ser21/Ser9) (Cell Signaling, cat. no. 9331), total GSK3 α/β (Cell Signaling, cat. no. 9832), phospho-MARCKS (Ser159/Ser163) (Cell Signaling, cat. no. 11992), total MARCKS (Proteintech cat. no. 20661), Vinculin (Cell Signaling, cat. no. 4650), PKCη (Abcam, cat. no. 179542), GAPDH (Cell Signaling, cat. no. 2118), Goat anti-mouse, Azure700 conjugate (Azure Biosystems, cat. no. AC2129), and Goat anti-rabbit, Azure800 conjugate (Azure Biosystems, cat. no. AC2134).

Phosphoproteomics

Sample preparations: The cerebellum of WT, HET, and HOM male and female mice at 6 months old was isolated, flash frozen in liquid nitrogen, and cryo-pulverized. Tissue was sent to the UCSD Biomolecular and Proteomics Mass Spectrometry Facility for Phosphoproteomic analysis (N=3 mice). Experimenter was blind to genotype and sex of samples. Samples were

lyophilized overnight and reconstituted in 200 µl of 6 M Guanidine-HCl. The samples were then boiled for 10 minutes followed by 5 minutes cooling at room temperature. The boiling and cooling cycle was repeated a total of 3 cycles. The proteins were precipitated with addition of methanol to final volume of 90% followed by vortex and centrifugation at maximum speed on a benchtop microfuge (14000 rpm) for 10 minutes. The soluble fraction was removed by flipping the tube onto an absorbent surface and tapping to remove any liquid. The pellet was suspended in 200 µl of 8 M Urea made in 100 mM Tris pH 8.0. TCEP was added to final concentration of 10 mM and Chloro-acetamide solution was added to final concentration of 40 mM and vortex for 5 minutes. 3 volumes of 50 mM Tris pH 8.0 were added to the sample to reduce the final urea concentration to 2 M. Trypsin (1:50 ratio) was incubated at 37°C for 12 hours. The solution was then acidified using TFA (0.5% TFA final concentration) and mixed. The sample was desalted using C18-StageTips (Thermo Fisher Scientific) as described by the manufacturer protocol. The peptide concentration of sample was measured using BCA. 80ug of each sample were used in TMT labeling. Thermo Scientific™ TMTpro™ 18plex (cat. no. A52045) labeling was carried out as described by the manufacturer. After labeling completion, the samples were pooled, and the peptides were desalted using 100 mg C18-SPR (waters) as described by the manufacturer protocol. The sample was lyophilized, and phospho-peptides were enriched using High-Select Fe-NTA Phosphopeptide Enrichment (cat. no. A32992, Thermo Fisher Scientific). The enriched phosphopeptide fraction was then further fractionated using Pierce™ High pH Reversed-Phase Peptide Fractionation Kit (Pierce™ High pH Reversed-Phase Peptide Fractionation Kit, cat. no. 84868) was used. Fractionation protocol as described by the manufacturer kit. Eight fractions were generated from this step and were analyzed as follows:

LC-MS-MS: 1 µg of each High pH Reversed-Phase enriched phosphopeptides was analyzed by ultra-high-pressure liquid chromatography (UPLC) coupled with tandem mass spectroscopy (LC-MS/MS) using nano-spray ionization. The FAIMS nano-spray ionization experiments were performed using an Orbitrap fusion Lumos hybrid mass spectrometer (Thermo

Fisher Scientific) interfaced with nano-scale reversed-phase UPLC (Thermo Dionex UltiMate™ 3000 RSLC nano System) using a 25cm, 75 µm ID glass capillary packed with 1.7 µm C18 (130) BEH™ beads (Waters corporation). Peptides were eluted from the C18 column into the mass spectrometer using a linear gradient (5–80%) of ACN (Acetonitrile) at a flow rate of 375 µl/minute for 120 minutes. The buffers used to create the ACN gradient were: Buffer A (98% H_2O, 2% ACN, 0.1% formic acid) and Buffer B (100% ACN, 0.1% formic acid). Mass spectrometer parameters are as follows; FAIMS CV setting: -40, -60, and -80V and carrier nitrogen gas flow set at 3.8 liters per minute. An MS1 survey scan using the orbitrap detector (mass range (m/z): 400-1500 (using quadrupole isolation), 60000 resolution setting, spray voltage of 2200 V, Ion transfer tube temperature of 290°C, AGC target of 400000, and maximum injection time of 50 ms) was followed by data dependent scans (top speed for most intense ions, with charge state set to only include +2-5 ions, and 5 second exclusion time, while selecting ions with minimal intensities of 50000 at in which the collision event was carried out in the high energy collision cell (HCD Collision Energy of 38%) and the first quadrupole isolation window was set at 0.8 (m/z). The fragment masses were analyzed in the orbitrap detector (mass range (m/z): automatic scan with first scan at m/z= 100). The resolution was set at 30000 resolutions. AGC Target set to 30000, and maximum injection time: 54 ms. Protein identification and quantification was carried out using Peaks Studio X (Bioinformatics solutions Inc.). Global Proteome analysis of TMT labelled sample: The flowthrough peptides from the phospho-peptide enrichment step were fractionated using Pierce™ High pH Reversed-Phase Peptide Fractionation Kit (Pierce™ High pH Reversed-Phase Peptide Fractionation Kit, cat. no. 84868). Fractionation protocol as described by the manufacturer kit.

Eight collected fractions were each analyzed by ultra-high-pressure liquid chromatography (UPLC) coupled with tandem mass spectroscopy (LC-MS/MS) using nano-spray ionization. The FAIMS nano-spray ionization experiments were performed using an Orbitrap fusion Lumos hybrid mass spectrometer (Thermo Fisher Scientific) interfaced with nano-scale reversed-phase UPLC (Thermo Dionex UltiMate™ 3000 RSLC nano System) using a 25 cm, 75 µm ID glass capillary

packed with 1.7 µm C18 (130) BEH^{TM} beads (Waters corporation). Peptides were eluted from the C18 column into the mass spectrometer using a linear gradient (5–80%) of ACN (Acetonitrile) at a flow rate of 375 µl/minute for 120 minutes. The buffers used to create the ACN gradient were: Buffer A (98% H_2O, 2% ACN, 0.1% formic acid) and Buffer B (100% ACN, 0.1% formic acid). Mass spectrometer parameters are as follows: FAIMS CV setting: -40, -60, and -80V and carrier nitrogen gas flow set at 3.8 liters per minute. A MS1 survey scan using the orbitrap detector (mass range (m/z): 400-1500 (using quadrupole isolation), 60000 resolution setting, spray voltage of 2200 V, Ion transfer tube temperature of 290°C, AGC target of 400000, and maximum injection time of 50 ms) was followed by data dependent scans (top speed for most intense ions, with charge state set to only include +2-5 ions, and 5 second exclusion time, while selecting ions with minimal intensities of 50000 at in which the collision event was carried out in the high energy collision cell (HCD Collision Energy of 38%) and the first quadrupole isolation window was set at 0.8 (m/z). The fragment masses were analyzed in the orbitrap detector (mass range (m/z): automatic scan with first scan at m/z = 100. The resolution was set at 30000 resolutions. AGC Target set to 30000, and maximum injection time: 54 ms. Protein identification and quantification was carried out using Peaks Studio X (Bioinformatics solutions Inc.).

Analysis: Identified phosphopeptides were normalized to their respective protein by dividing the intensity of the phosphopeptide by the intensity of its corresponding protein per sample. Phosphopeptides were removed if the intensity of the phosphopeptide or the intensity of its corresponding protein were below detection in a sample in a given comparison, or if phosphopeptides were ambiguously aligned to a protein. Comparisons were made between WT females vs HET females, WT females vs HOM females, WT males vs HET males, WT males vs HOM males, HET females vs HET males, and HOM females vs HOM males. Significance was determined by student's *t*-test and fold change was calculated from the normalized mean intensity. A p-value of 0.05 was selected to identify global trends in pathway alteration. Volcano plots show log-transformed p-values vs the log-transformed fold change quantified per change in normalized

phosphopeptide abundance between comparisons. Next, Ontology enrichment analysis was conducted using EnrichR package (R package version 3.2, 2023) after using the BiomaRt package (R package version 2.58.2) to assign entrez gene names (6-8). Graphs display the top ten most significant ontologies for GO Biological Process 2023, GO Cellular Component 2023, and GO Molecular Function 2023 from the Gene Ontology Consortium(9, 10) ($p<0.05$). Additionally, heatmaps were generated to display phosphopeptide changes that trend (increase or decrease) with increasing ΔF48 alleles. These were selected by identifying normalized phosphopeptides significantly altered in HOM compared to WT with a corresponding trend (or significant change) in HET compared to WT for each sex. Heatmaps were also created for phosphopeptide changes that trend (increase or decrease) similarly in both male and female mice in WT vs HET or WT vs HOM. These similarly change phosphopeptides are defined as having the same trend in males and females with significant differences in normalized phosphopeptide abundance between WT vs HET or WT vs HOM in at least one sex. Analysis, statistics, and graphical representations were all performed in R programming (R version 4.3.3; R studio Version 2023.12.1+402)(11).

Kinase enrichment analysis

Kinase activity inference was performed using The Kinase Library Motif Enrichment Analysis (MEA) algorithm, a permutation-based enrichment framework for quantifying motif-level regulation from phosphoproteomic data (12, 13). For each kinase, the substrate list was defined as all phosphosites for which that kinase ranked among the top-15 kinases based on the motif–substrate percentile scores derived from The Kinase Library atlas. Within each dataset, phosphosites were ranked according to the composite statistic −sign(log2FC)*log10(p-value), such that highly upregulated and statistically significant sites appeared at the top of the list and downregulated sites at the bottom. Enrichment was evaluated by traversing the ranked list and calculating a running-sum statistic that increased when a phosphosite belonged to the kinase-

specific list and decreased otherwise. The resulting enrichment score (ES) quantifies the degree to which regulated phosphosites are overrepresented among predicted substrates of a given kinase. Normalized enrichment scores (NES) and empirical p-values were obtained by random permutation of site labels to generate null distributions. The enrichment analysis was implemented using the GSEApy Python package(14).

Statistical Analysis

Statistical comparisons were made using one-way analysis of variance (ANOVA), Student's *t*-test, or χ^2-test where indicated. Sex was considered a biological variable and all tests utilized male and female mice. No age-related differences were observed between the ages of mice tested, therefore, data from age-matched mouse cohorts of different ages were combined if applicable after normalization to the WT group within each age-matched cohort. All groups (sex and genotype) were present in each cohort when tested. Outliers were determined using Grubbs' test (α=0.05) where appropriate. All bar plots are shown as mean±S.E.M. Analysis, statistics, and graphical representations were prepared in R programming (R version 4.3.3; R studio Version 2023.12.1+402)(11). Protein interaction network was generated using STRING (version 12)(15).

Supplemental References

1. Behera S, et al. Comprehensive genome analysis and variant detection at scale using DRAGEN. *Nat Biotechnol.* 2025;43(7):1177-91.
2. Danecek P, et al. Twelve years of SAMtools and BCFtools. *Gigascience.* 2021;10(2).
3. Robinson JT, et al. Variant Review with the Integrative Genomics Viewer. *Cancer Res.* 2017;77(21):e31-e4.
4. Schindelin J, et al. Fiji: an open-source platform for biological-image analysis. *Nat Methods.* 2012;9(7):676-82.
5. Rüesgsegger THaP. A new method for the model-independent assessment of thickness in three-dimensional images. *Journal of Microscopy.* 1996(185):67-75.
6. Chen EY, et al. Enrichr: interactive and collaborative HTML5 gene list enrichment analysis tool. *BMC Bioinformatics.* 2013;14:128.
7. Kuleshov MV, et al. Enrichr: a comprehensive gene set enrichment analysis web server 2016 update. *Nucleic Acids Res.* 2016;44(W1):W90-7.
8. Xie Z, et al. Gene Set Knowledge Discovery with Enrichr. *Curr Protoc.* 2021;1(3):e90.
9. Ashburner M, et al. Gene ontology: tool for the unification of biology. The Gene Ontology Consortium. *Nat Genet.* 2000;25(1):25-9.
10. Gene Ontology C, et al. The Gene Ontology knowledgebase in 2023. *Genetics.* 2023;224(1).
11. RCoreTeam. R: A Language and Environment for Statistical Computing. *R Foundation for Statistical Computing, Vienna, Austria.* 2024.
12. Yaron-Barir TM, et al. The intrinsic substrate specificity of the human tyrosine kinome. *Nature.* 2024;629(8014):1174-81.
13. Johnson JL, et al. An atlas of substrate specificities for the human serine/threonine kinome. *Nature.* 2023;613(7945):759-66.
14. Fang Z, et al. GSEApy: a comprehensive package for performing gene set enrichment analysis in Python. *Bioinformatics.* 2023;39(1).
15. Szklarczyk D, et al. The STRING database in 2023: protein-protein association networks and functional enrichment analyses for any sequenced genome of interest. *Nucleic Acids Res.* 2023;51(D1):D638-D46.

Supplemental Figures

A

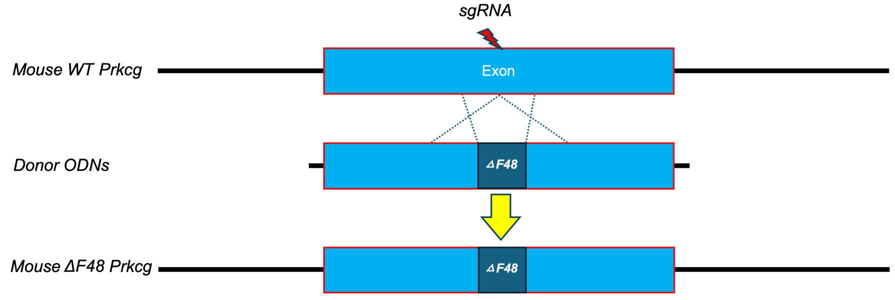

B

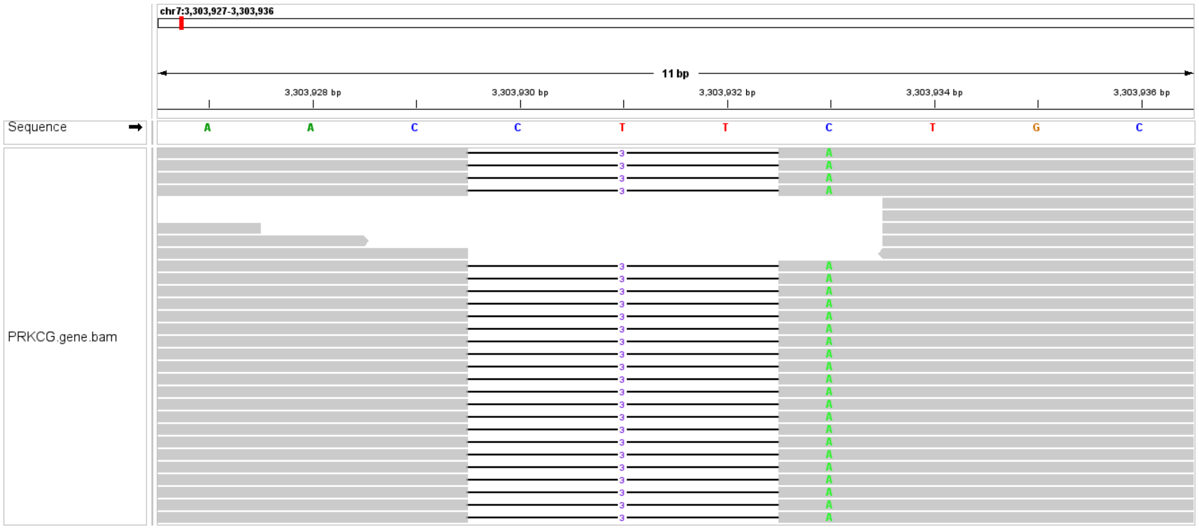

C

Off Target Candidates:	Genome Location	Reference Allele (Ref)	WT Sequence	HOM Sequence	WT-HOM Match	WT-Ref Match	HOM-Ref Match
**Candidate 1** Gene: chrna9	chromosome:GRCm39:5:66125526:66125554:1	GTTAGTGCAGTGACTGCAGAAGCCGG	GTTAGTGCAGTGACTGCAGAAGCCGG	GTTAGTGCAGTGACTGCAGAAGCCGG	TRUE	TRUE	TRUE
**Candidate 2** Gene: Prkca	chromosome:GRCm39:11:108234329:108234355:1	GTCGGTGCAGTGGCTGCAGAAGGTGG	GTCGGTGCAGTGGCTGCAGAAGGTGG	GTCGGTGCAGTGGCTGCAGAAGGTGG	TRUE	TRUE	TRUE
**Candidate 3** Gene: gm17733	chromosome:GRCm39:6:115056879:115056905:-1	CCTTCTTCTGCAGACACTGTACGTAC	CCTTCTTCTGCAGACACTGTACGTAC	CCTTCTTCTGCAGACACTGTACGTAC	TRUE	TRUE	TRUE
**Candidate 4** Gene: prkcb	chromosome:GRCm39:7:121888707:121888733:-1	CCACCTTCTGCAGCCACTGCACCGAC	CCACCTTCTGCAGCCACTGCACCGAC	CCACCTTCTGCAGCCACTGCACCGAC	TRUE	TRUE	TRUE
**Candidate 5** Gene: Gm32255	chromosome:GRCm39:10:67367101:67367127:1	GTCTCTACACTGACTGCAGAGGGTGG	GTCTCTACACTGACTGCAGAGGGTGG	GTCTCTACACTGACTGCAGAGGGTGG	TRUE	TRUE	TRUE
**Candidate 6**	chromosome:GRCm39:19:6805886:6805912:1	GTCTGCACAGTGCCTGCAGTAGGAGG	GTCTGCACAGTGCCTGCAGTAGGAGG	GTCTGCACAGTGCCTGCAGTAGGAGG	TRUE	TRUE	TRUE

D

WT PKCγ (700bp)

5'–AGGTGCTGAGAGCGAAGCTCCCGCCGCCGCCCGTGCCTGCGGCTCCTTGATGCCCCAGCCTTCAGCTCTGA
CCCCACCCGCTTTCTCCCCGGTCGGTGCTGCCCGTGCCGGGGTGTTGCTTTCTGCCGTGTGCTGTGCACCGTT
AGGTGCCTTGCCCCTGTCCTTCCTATCTCAGAGTCTGCGGAGTCCTCCTACCGCCGTCCACCTGTTTCCTCGGA
AAAAGGGCCAGCTCGTGATCCCTTCTGCGTTCCTGGGGCCATGGCGGGTCTGGGCCCTGGCGGAGGCGACTC
AGAGGGGGGACCCCGACCCCTGTTTTGCAGAAAGGGGGCGCTGAGGCAGAAGGTGGTCCACGAGGTGAAGAG
CCACAAGTTCACCGCTCGTTTCTTCAAGCAGCCAACC**TTC**TGCAGTCACTGTACCGACTTCATCTGGTGAGGGAA
GCGGGCTAGGGGAGAGGGCTGGAAAAGGAGGGGACTTGGGAACAGTCATGTCACACTGGTCCCCAAGCGACT
GAGCAGAGAGGGGCTGCAGCTCCCACTCCTGGGCTTAATGGCAGGGGGTGGAAGTCTGGGTTCCTGGGTCTG
AGGGAGGAAGAGCCAGGCTGGATTCTAGGGTCCAAGGGAAAGGGAGCTGGGACTAGAAATTTTGGGTTCTCGG
AGAAGGCAACTAGGACTCAGACATCTGGGTGAAATTGGGAAACTGC–3'

ΔF48 PKCγ (Restriction enzyme digest = 300 bp and 400 bp)

5'–**AGGTGCTGAGAGCGAAGC**TCCCGCCGCCGCCCGTGCCTGCGGCTCCTTGATGCCCCAGCCTTCAGCTCTG
ACCCCACCCGCTTTCTCCCCGGTCGGTGCTGCCCGTGCCGGGGTGTTGCTTTCTGCCGTGTGCTGTGCACCGT
TAGGTGCCTTGCCCCTGTCCTTCCTATCTCAGAGTCTGCGGAGTCCTCCTACCGCCGTCCACCTGTTTCCTCGG
AAAAAGGGCCAGCTCGTGATCCCTTCTGCGTTCCTGGGGCCATGGCGGGTCTGGGCCCTGGCGGAGGCGACT
CAGAGGGGGGACCCCGACCCCTGTTTTGCAGAAAGGGGGCGCTGAGGCAGAAGGTGGTCCACGAGGTGAAGA
GCCACAAGTTCACCGCTCGTTTCTTCAAGCAGCCA**ACATGC**AGTCACTGTACCGACTTCATCTGGTGAGGGAAG
CGGGCTAGGGGAGAGGGCTGGAAAAGGAGGGGACTTGGGAACAGTCATGTCACACTGGTCCCCAAGCGACTG
AGCAGAGAGGGGCTGCAGCTCCCACTCCTGGGCTTAATGGCAGGGGGTGGAAGTCTGGGTTCCTGGGTCTGA
GGGAGGAAGAGCCAGGCTGGATTCTAGGGTCCAAGGGAAAGGGAGCTGGGACTAGAAATTTTGGGTTCTCGGA
GAAGGCAACTAGGACTCAGACATCT**GGGTGAAATTGGGAAACTGC**–3'

E

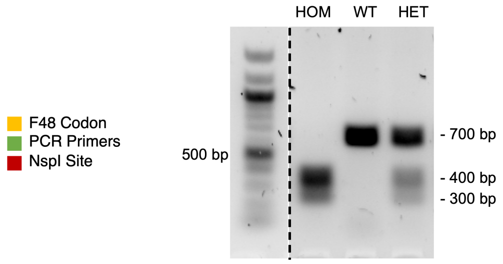

Supplemental Figure 1: Generating SCA14 mice using CRISPR/Cas9-mediated genome editing method

A) Schematic of the genome editing strategy to incorporate the donor sequence with deleted Phe48 (ΔF48) in PKCγ. Whole genome sequencing was performed on a WT and HOM mouse to B) validate the incorporation of ΔF48 in the PKCγ gene, and to C) determine that no unintended mutations were introduced in genomic regions with sequence similarity by assessing candidate locations of potential off-target sites. D) All mice were genotyped using the depicted strategy. PCR was performed on gDNA to amplify a 700bp section of the *Prkcg* gene (F48 codon shown in yellow). Primers were designed to anneal to the sequence shown in green. Mice containing the ΔF48 PKCγ donor sequence have a Nsp1 restriction enzyme cut site (red) while WT mice do not, therefore, restriction enzyme digest will cut ΔF48 PKCγ producing two smaller bands (300 and 400bp) and not WT PKCγ (700bp). E) DNA gel represents genotyping results for WT, HET, and HOM mice. WT mice have one 700bp band; HET have three bands at 700, 300, and 400bp; and HOM mice have two bands at 300, and 400bp.

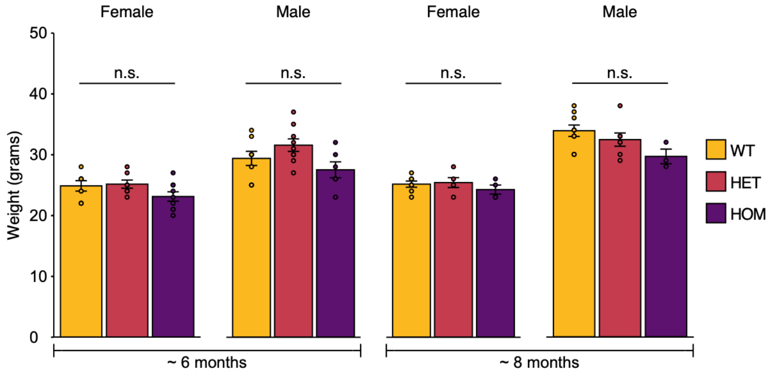

Supplemental Figure 2: WT and SCA14 mouse weights

To assess health of SCA14 mice, the weight of all genotypes (WT, HET, HOM) and sexes were measured, and no physical abnormalities or survival issues were noted in adult animals post-development. Representative weights are shown for a cohort measured at 6 months and 8 months. HOM mice tend to be slightly smaller, but not significantly less. Bar graphs represented quantification of mean±S.E.M. Significance was determined by One-way ANOVA with Tukey's post hoc (not significant indicated by n.s.).

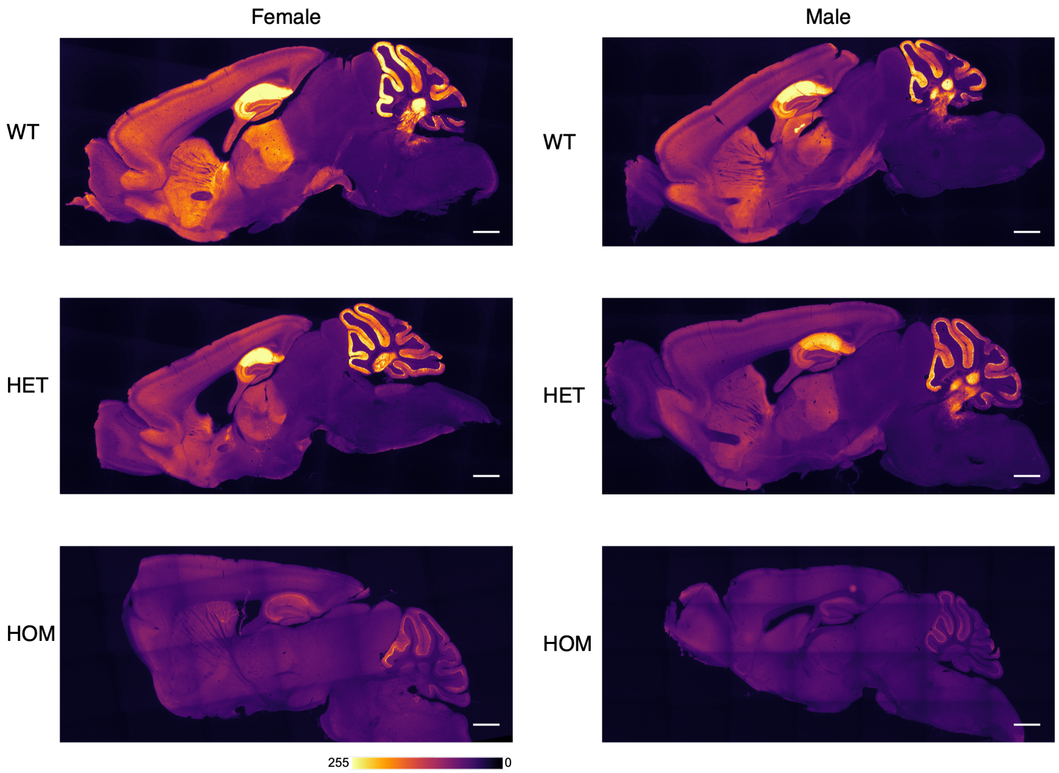

Supplemental Figure 3: PKCγ is reduced in whole brain of SCA14 mice

Fluorescence immunohistochemistry of PKCγ was performed on fixed sagittal brain sections (40μm) from SCA14 mice of all genotypes (WT, HET, HOM) and sexes, and imaged using widefield microscopy on a Keyence Automate Imaging System microscope (Keyence Corporation). Representative images of PKCγ staining indicate a gene dose dependent loss of PKCγ in whole brain (Estimated scale bar=1mm, respective color scale indicates intensity).

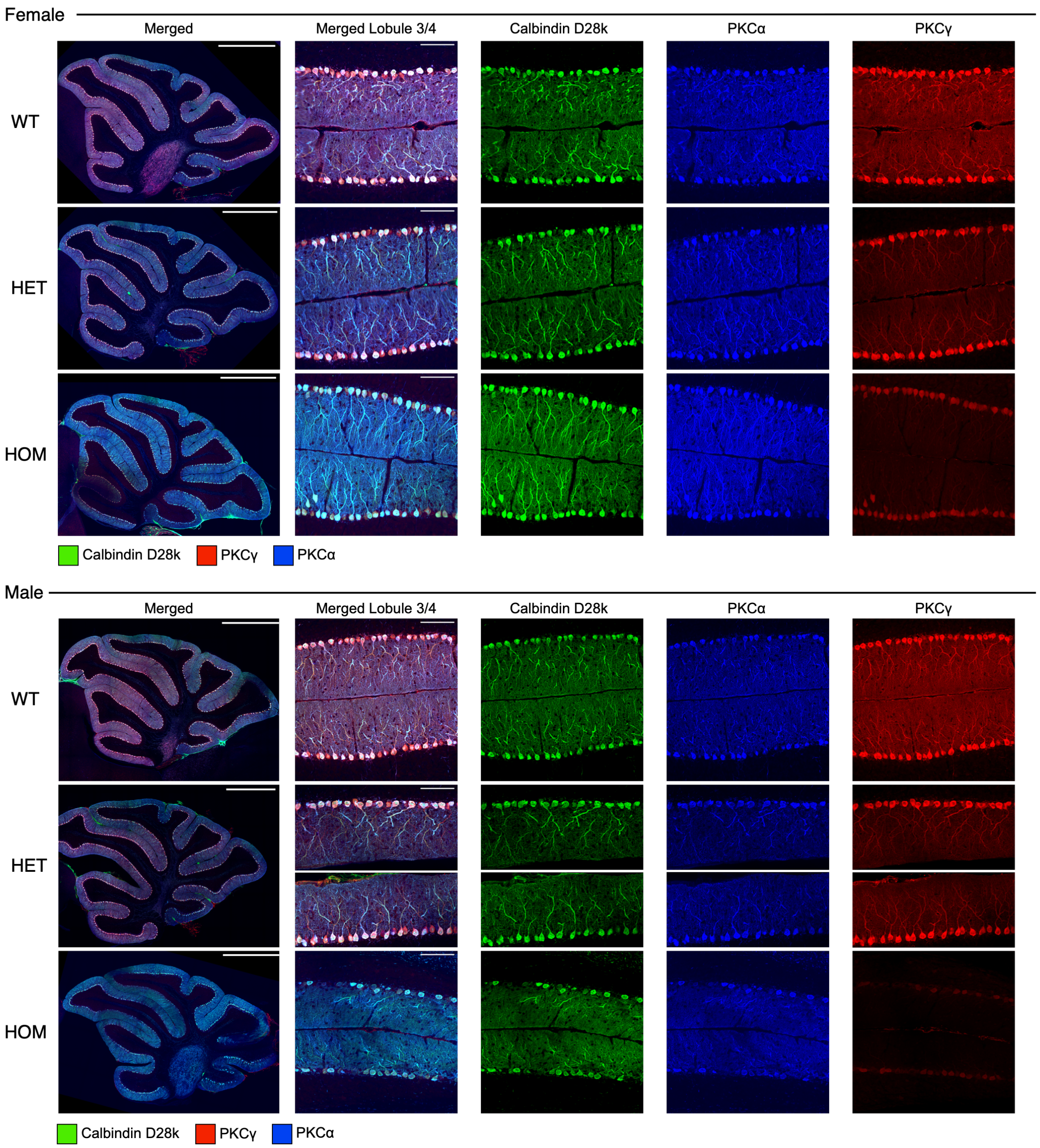

Supplemental Figure 4: PKCγ and PKCα are localized to Purkinje cells of the Cerebellum Fluorescence immunohistochemistry was performed on fixed sagittal brain sections from all genotypes (WT, HET, HOM) and sexes. Shown are unquantified max projected images to allow visualization of the cerebellum and colocalization of Calbindin D28k (green), PKCγ (red), and PKCα (blue) in the whole cerebellum and a magnification of the PCL and ML in lobule 3 and 4 as a merged image as well as for each protein individually. Whole cerebellum scale bar=1mm, PCL and ML magnification scale bar=100μm.

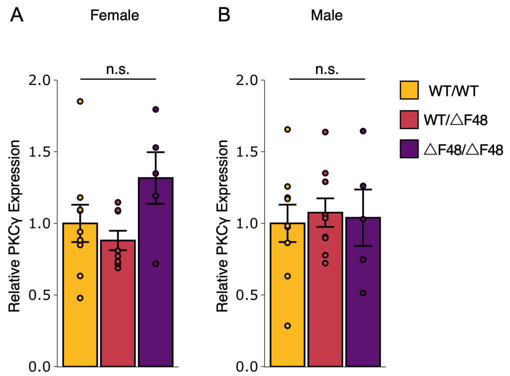

Supplemental Figure 5: SCA14 mice have equivalent levels of PKCγ RNA in the cerebellum

RT-qPCR was performed on RNA isolated from whole cerebellar homogenate from all genotypes (WT, yellow; HET, red; HOM, purple) and sexes to assess transcriptional changes. No significant changes in PKCγ RNA abundance were detected in A) female or B) male mice. The ΔΔCt method was used to quantify relative abundance and data is normalized to WT control group (N=5-9 mice per group). Bar graphs represented quantification of mean±S.E.M. Significance was determined by One-way ANOVA with Tukey's post hoc (not significant indicated by n.s.).

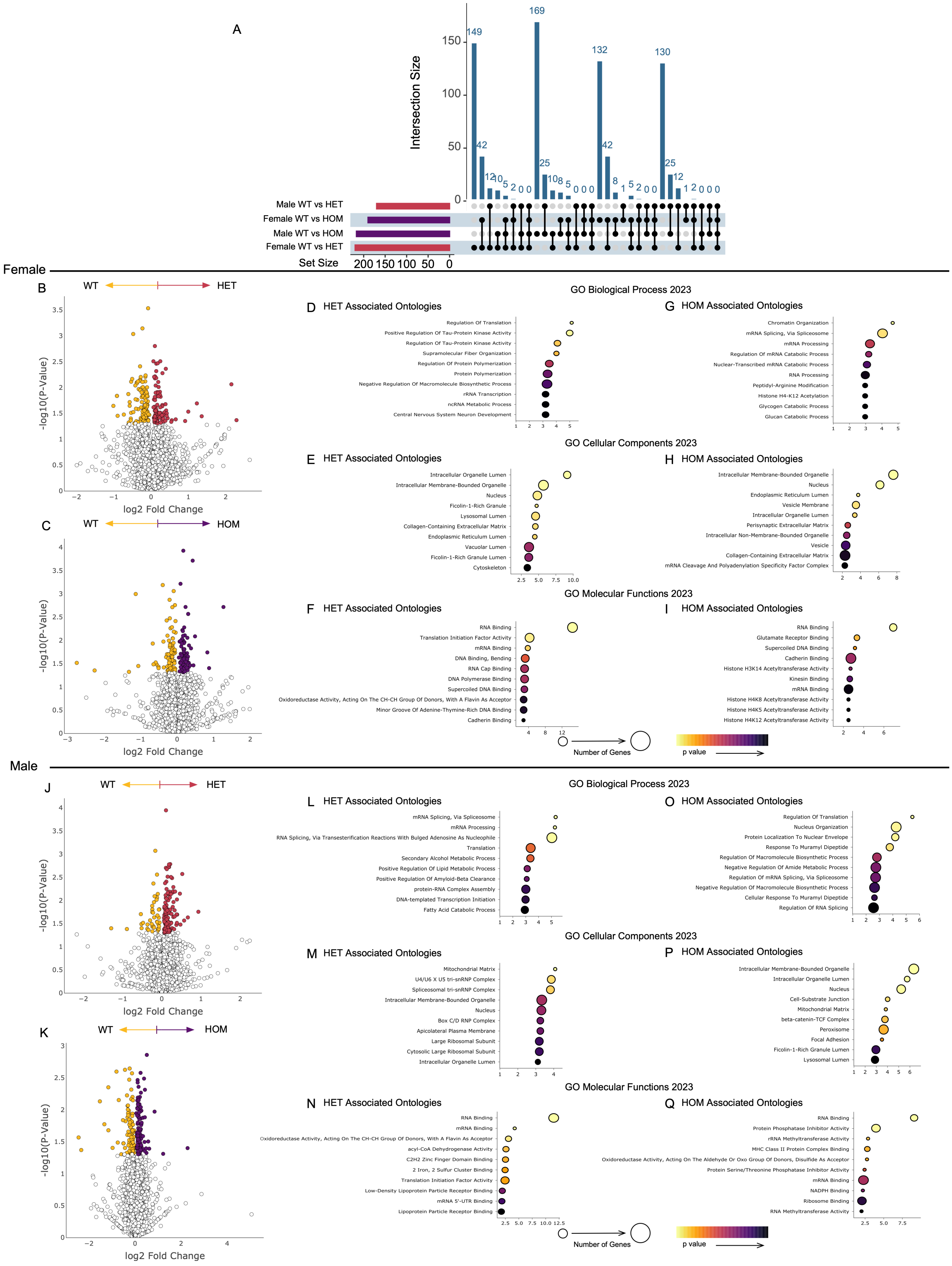

Supplemental Figure 6: ΔF48 PKCγ alters the cerebellar proteome in SCA14 mice

Proteomic analysis was carried out on protein extracted from whole cerebellar homogenate from all genotypes (WT, yellow; HET, red; HOM, purple) and sexes (N=3 mice per group). A total of 5216 quantifiable proteins were detected. A) An upset plot summarizes the quantity of significantly different proteins and shared proteins identified between groups, with male HOM mice exhibiting the largest difference in the proteome compared to WT mice ($p<0.05$). Volcano plots show log-transformed p-values vs the log-transformed fold change quantified per change in protein abundance between WT compared to B) HET and C) HOM in female mice. Color represents phosphopeptides with p-value < 0.05. Dot plots indicate the top 10 most significantly enriched for gene ontologies from the differentially abundant proteins identified in D-F) WT vs HET and G-I) WT vs HOM females for biological processes, cellular components and molecular functions. Volcano plots show log-transformed p-values vs the log-transformed fold change quantified per change in protein abundance between WT compared to J) HET and K) HOM in male mice. Color represents phosphopeptides with p-value < 0.05. Dot plots indicate the top 10 most significantly enriched for gene ontologies from the differentially abundant proteins identified in L-N) WT vs HET and o-q) WT vs HOM males for biological processes, cellular components and molecular functions. Dot plots show ontology vs -log10(p-value), and plot color scale indicates p-value while dot size indicates number of genes per ontology ($p<0.05$).

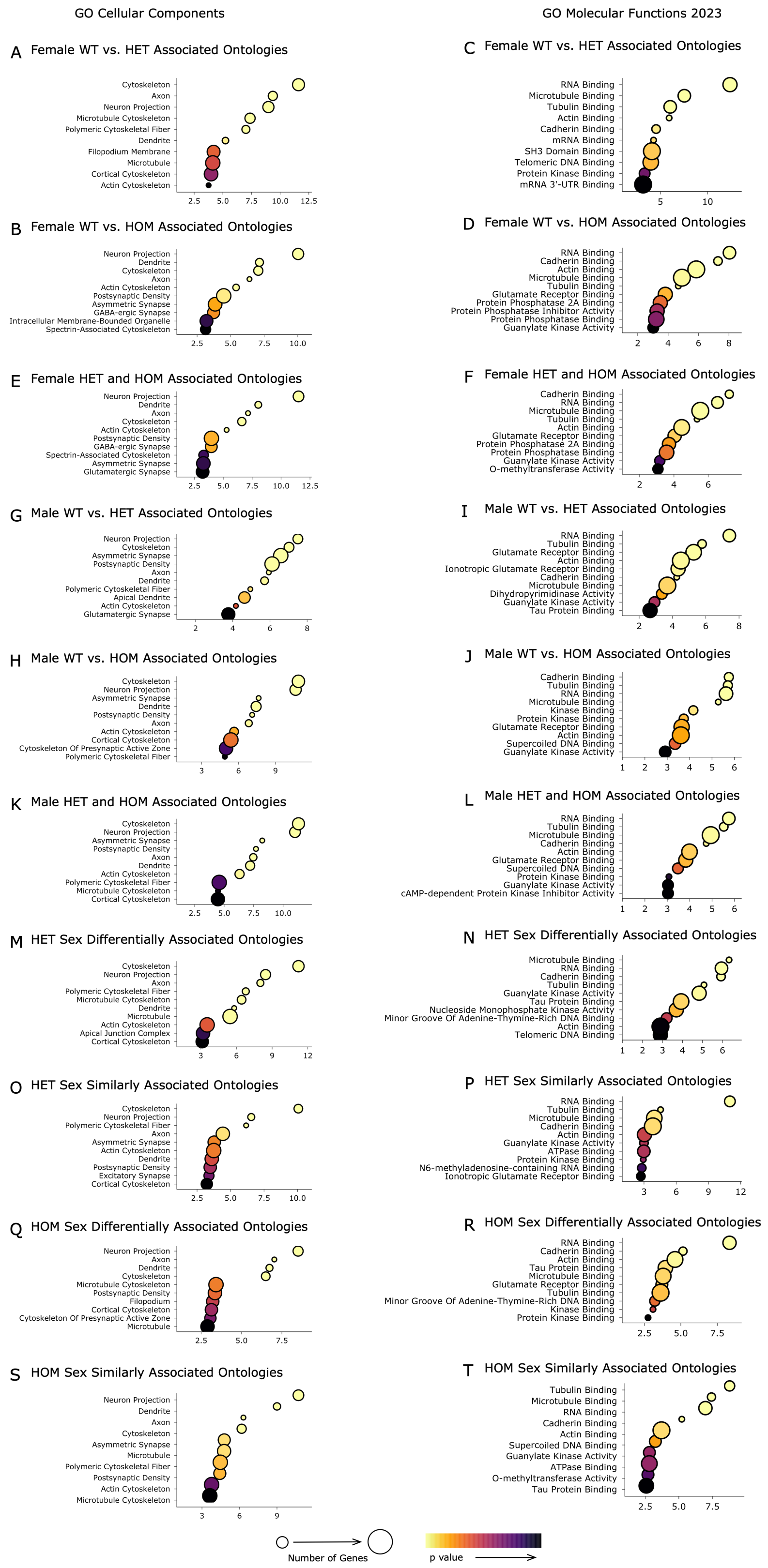

Supplemental Figure 7: Ontology analysis of the SCA14 mouse phosphoproteome

Phosphoproteomic analysis was carried out on protein extracted from whole cerebellar homogenate from all genotypes (WT, HET, HOM) and sexes (N=3 mice per group). Changes in phosphopeptide intensity were normalized to the corresponding protein intensity. Significant differences in phosphopeptides abundance (as determined by two sample t-test <0.05) were compared between WT vs HET and WT vs HOM in female and male mice, or male vs female in HET and HOM mice. Gene ontology analysis was performed, and graphs indicate the top ten most significant ontologies for Cellular components (left) and Molecular functions (right) determined with EnrichR. Dot plots show ontology vs -log10(p-value), and plot color scale indicates p-value while dot size indicates number of genes per ontology ($p<0.05$).

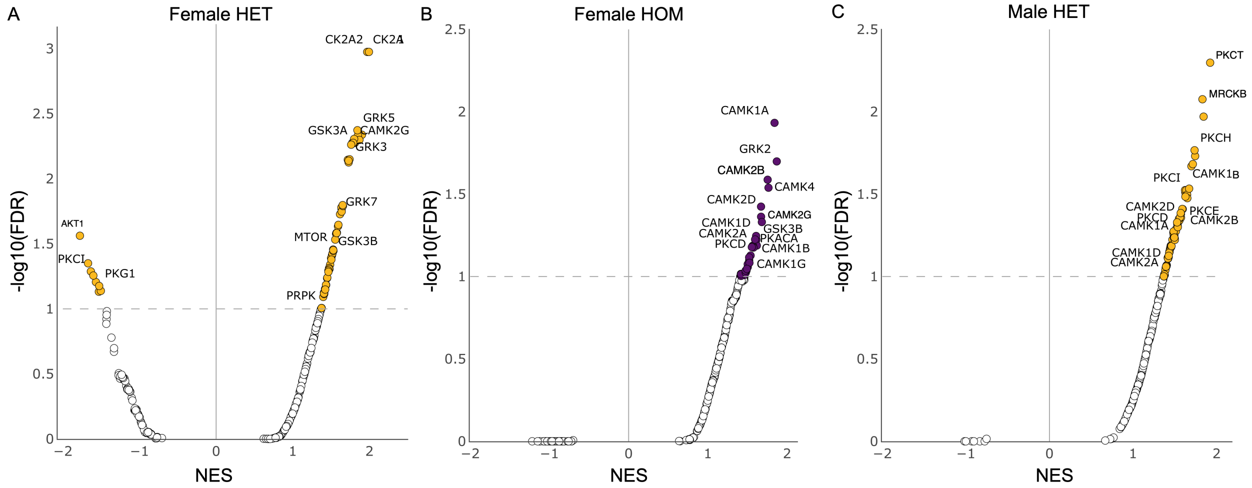

Supplemental Figure 8: Kinase motif enrichment analysis of the rewired phosphoproteome in male and female SCA14 genotypes

Kinase motif enrichment analysis of identified phosphopeptides in A) WT vs HET female, B) WT vs HOM female, and C) WT vs HET male phosphoproteomic comparisons revealed upregulated motifs corresponding to multiple kinases. Color indicates significance (FDR < 0.1).
