# Supplementary material for: Sex-specific disruptions in PKCγ signaling in a mouse model of spinocerebellar ataxia type 14
Source: JCI Insight. 2026 Apr 2;11(10):e192155. doi: 10.1172/jci.insight.192155 (PMC13232732; doi:10.1172/jci.insight.192155)

Figure 3A Left

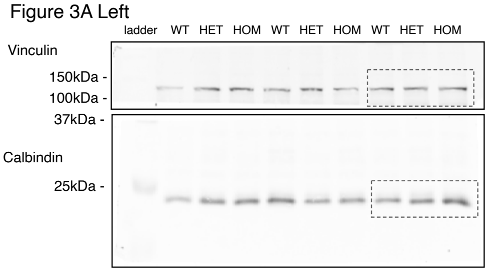

Figure 3A Right

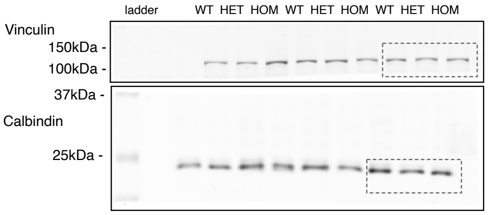

Figure 5A Left

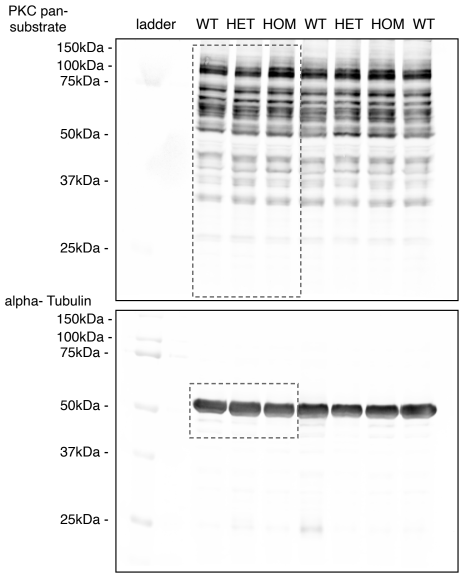

Figure 5D Left

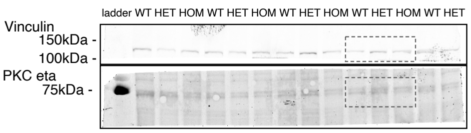

Figure 6B Left

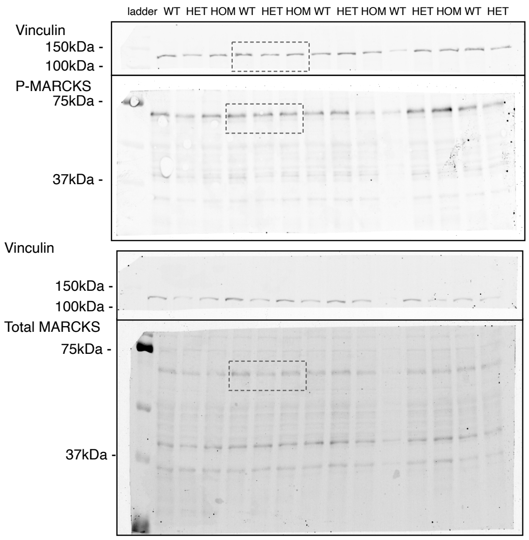

Figure 4A Left

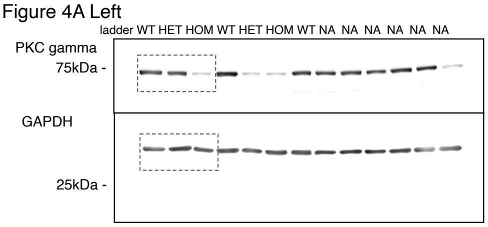

Figure 4A Right

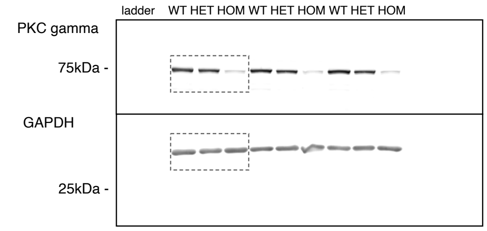

Figure 5A Right

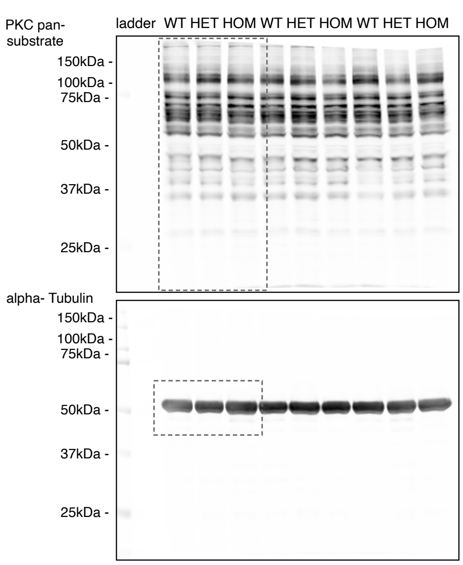

Figure 5D Right

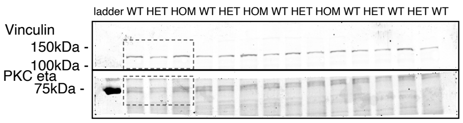

Figure 6B Right

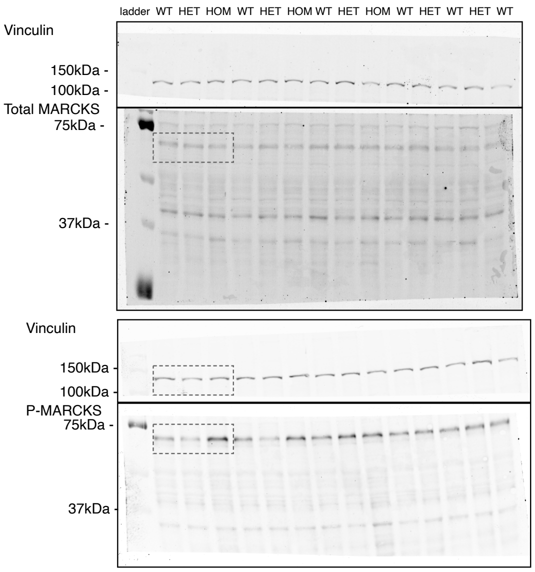

Figure 4B Left

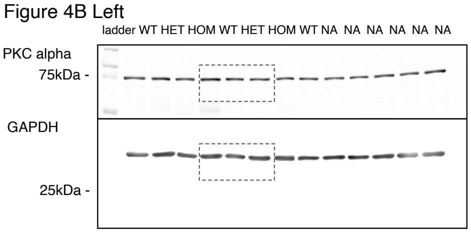

Figure 4B Right

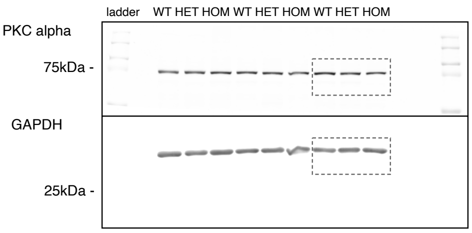

Figure 5B Left

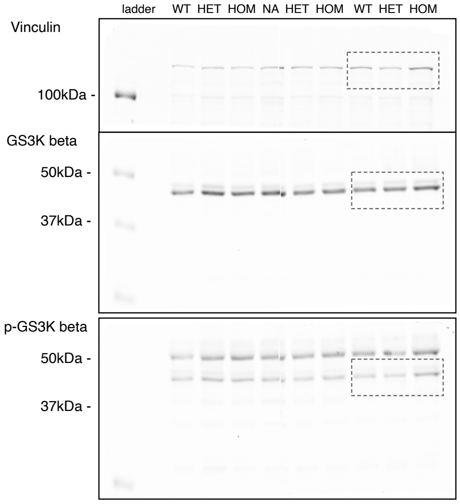

Figure 5B Right

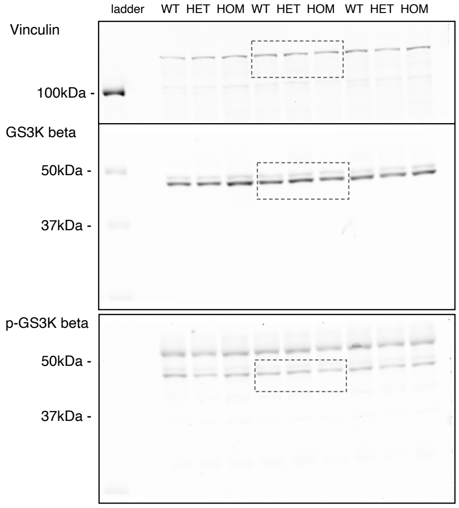

Figure 5C Left

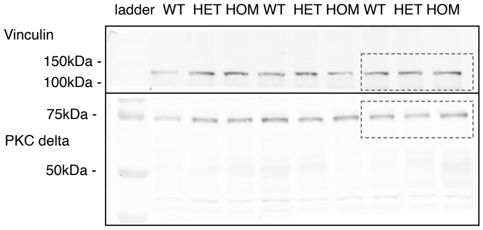

Figure 5C Right

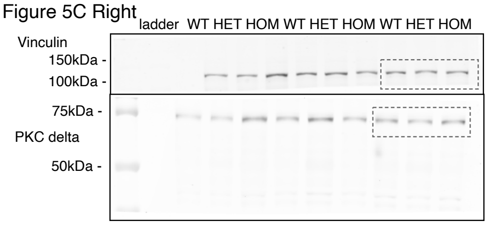

Supplement: Unedited blot and gel images [file jciinsight-11-192155-s225.pdf]
